# Global patterns of climate change impacts on desert bird communities

**DOI:** 10.1038/s41467-023-35814-8

**Published:** 2023-01-13

**Authors:** Liang Ma, Shannon R. Conradie, Christopher L. Crawford, Alexandra S. Gardner, Michael R. Kearney, Ilya M. D. Maclean, Andrew E. McKechnie, Chun-Rong Mi, Rebecca A. Senior, David S. Wilcove

**Affiliations:** 1 Princeton School of Public and International Affairs, Princeton University, Princeton, NJ USA; 2 School of Ecology, Shenzhen Campus of SunYat-sen University, Shenzhen, Guangdong People’s Republic of China; 3 South African Research Chair in Conservation Physiology, South African National Biodiversity Institute, 2 Cussonia Ave, Brummeria, Pretoria 0184 South Africa; 4 DSI-NRF Centre of Excellence at the FitzPatrick Institute, Department of Zoology and Entomology, University of Pretoria, Lynnwood Rd., Pretoria, 0002 South Africa; 5 Environment and Sustainability Institute, University of Exeter Penryn Campus, Penryn, Cornwall TR10 9FE UK; 6 School of BioSciences, The University of Melbourne, Melbourne, VIC 3010 Australia; 7 Key Laboratory of Animal Ecology and Conservation Biology, Institute of Zoology, Chinese Academy of Sciences, Beijing, People’s Republic of China; 8 Conservation Ecology Group, Department of Biosciences, Durham University, Durham, DH1 3LE UK; 9 Department of Ecology and Evolutionary Biology, Princeton University, Princeton, NJ USA

**Keywords:** Conservation biology, Climate-change ecology, Biodiversity, Ecological modelling

## Abstract

The world’s warm deserts are predicted to experience disproportionately large temperature increases due to climate change, yet the impacts on global desert biodiversity remain poorly understood. Because species in warm deserts live close to their physiological limits, additional warming may induce local extinctions. Here, we combine climate change projections with biophysical models and species distributions to predict physiological impacts of climate change on desert birds globally. Our results show heterogeneous impacts between and within warm deserts. Moreover, spatial patterns of physiological impacts do not simply mirror air temperature changes. Climate change refugia, defined as warm desert areas with high avian diversity and low predicted physiological impacts, are predicted to persist in varying extents in different desert realms. Only a small proportion (<20%) of refugia fall within existing protected areas. Our analysis highlights the need to increase protection of refugial areas within the world’s warm deserts to protect species from climate change.

## Abstract (web summary)

Desert-dwelling species are adapted to high temperatures, but further warming may push them beyond their physiological limits. Here, the authors integrate biophysical models and species distributions to project physiological impacts of climate change on desert birds globally and identify potential refugia.

## Introduction

Climate change is causing major shifts in the distributions and abundances of species around the world^1–3^. However, comparatively little attention has been paid to the impacts of climate change on desert ecosystems, and the few studies that exist focus on either polar regions^4,5^ or deserts in a few countries^6–8^. This lack of attention to the world’s warm deserts is concerning because: (1) warm deserts harbor surprisingly high levels of biodiversity^9^, including many species that do not occur elsewhere^10^; (2) deserts have already felt the impacts of climate change more than most other ecosystems^11^ and are predicted to experience considerable increases in absolute temperature in the future^12^; and (3) many desert species already live close to their physiological limits^13^. Thus, further climate change could push desert species beyond these limits^14^.

As such, it is important to be able to predict how climate change will affect desert species. Understanding the spatial patterns of predicted temperature change is an essential first step. However, simply mapping air temperature change may provide an incomplete picture of how species will be affected, given that species operate in microclimates^15^ which, together with their behavioral and physiological responses, may buffer or magnify the impacts of climate change to varying extends^16^. As a result, physiological models using microclimatic forcing data should offer more realistic assessments of how desert species will likely respond to climate change^17^.

Here, we combine a microclimate model and a physiologically explicit biophysical model with climate change projections and biodiversity maps to address the following questions: (a) How will the world’s warm deserts be affected by climate change, and do the projected impacts vary between and within major desert realms? (b) Does a physiological model of climate change impacts on desert birds produce spatially different results from models based solely on air temperature (Tair)? (c) Which areas within each of the world’s warm deserts are likely to serve as refugia for desert birds in the face of climate change? (d) To what extent do these refugia fall within the boundaries of existing protected areas (PAs)?

We focus on birds because of their diurnality and limited ability to use thermally buffered microsites such as burrows^18–20^, which makes them particularly exposed to extreme climates relative to other taxa. Additionally, birds have among the highest mass-specific evaporative water loss rates of any terrestrial animals, which may render them more sensitive to further warming^13,21^. Indeed, researchers have already documented major declines in avian abundance in some desert regions as a result of climate change^22–24^.

Of the various aspects of avian physiology that are potentially sensitive to climate change, water balance is critically important to desert birds because of the trade-off between maintaining body temperatures below lethal limits by increasing evaporative water loss and avoiding dehydration^13,25^. Thus, we use two physiological metrics of climate change impact: total evaporative water loss (TEWL; i.e., water loss in an average day of the typically hottest month of the year) and acute dehydration risk (ADR; i.e., maximum water loss per gram of mass in three continuous hours in an average day of the typically hottest month of the year), which have been shown to determine species’ likelihood of surviving under long-term warming^20,26^ and extreme heat waves, respectively^13,19,27^. We focus on two future climate change scenarios in which global mean temperatures are, on average, 2 °C (main text) and 4 °C warmer (Supplementary Information) than pre-industrial values. The climate projections account for geographic patterns in warming and for changes in radiation, humidity, and wind speed. Conclusions hold for both scenarios. We found that heterogeneity in predicted climate change impacts exist both between and within major warm deserts. The physiological model of climate change impacts produced spatially different results from models based solely on air temperature. Most identified climate change refugia, which were the areas with high desert bird diversity and low climate change impact, lie close to coastlines. Only a very small proportion of identified refugia fall within the borders of existing PAs.

## Results

### Heterogeneity in predicted climate change impacts on desert birds

**Fig. 1:**
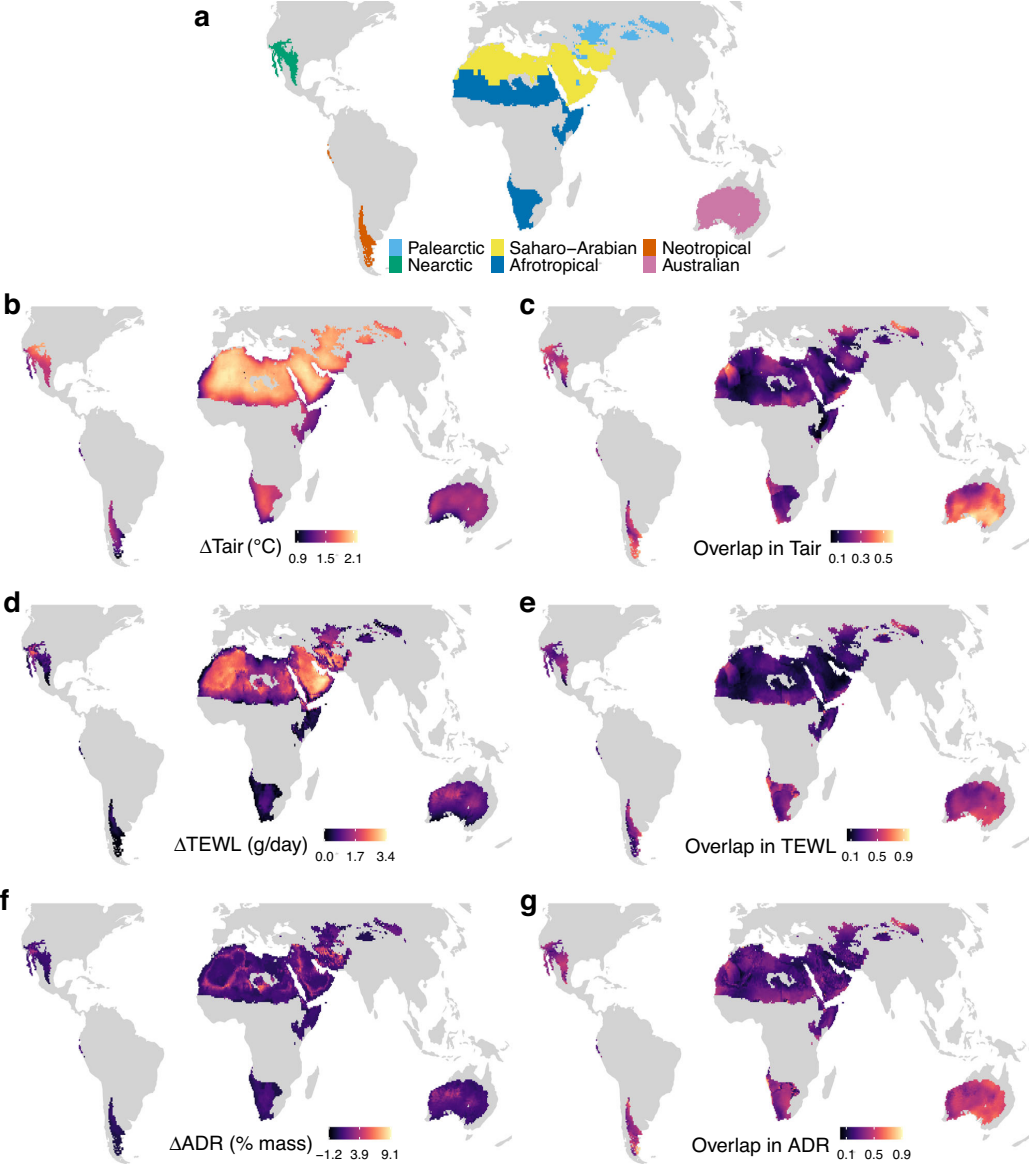
Climate change impacts for desert birds when global mean temperatures are 2 °C warmer than pre-industrial values. The climate change impacts are shown as estimated changes in mean values (panels **b**, **d**, and **f**; “Δ” represent value changes; warmer colors indicate higher impact) and proportion of overlap between current and future values (panels **c**, **e**, and **g**; cooler colors indicate higher impact) of air temperature (Tair; °C), total evaporative water loss (TEWL; g/day) and acute dehydration risk (ADR; percent of body mass) during the hottest month (July for Northern Hemisphere, January for Southern Hemisphere). Panel **a** shows the locations of the six major realms containing warm deserts (“desert realms”, represented by colors) and desert birds (bird species having ≥90% of their habitat within warm deserts). We assumed that a bird actively shifts between open and shaded habitat to minimize its rate of water loss. See Supplementary Fig. 6 for climate change impacts estimated assuming a bird always stays in the open. See Supplementary Figs. 7, 8 for results for a scenario in which global mean temperatures are 4 °C warmer than pre-industrial values.

Our analysis, based on three model species representing desert birds that fall within three size categories, revealed considerable heterogeneity in predicted climate change impacts on birds between and within global warm deserts (Fig. 1). According to climate models and our projections, the largest change in mean values of air temperature (Tair) and TEWL will occur in the Saharo-Arabian desert realm, while that of ADR is similar among desert realms (desert realm locations shown in Fig. 1a). We estimated the “proportion of overlap” (overlapping area of kernel density estimations) between current (1986–2015) and future values of Tair, TEWL and ADR, as it considers not only the change in mean but also the overall variance between years. The smallest proportion of overlap for Tair, TEWL, and ADR occurs in the Saharo-Arabian desert realm (Supplementary Fig. 4; *p* < 0.001). We used the proportion of overlap between current and future values of the two physiological metrics (TEWL and ADR) to represent climate change impact (less overlap means larger impact) hereafter. The probability distributions of climate change impact vary between desert realms (Supplementary Fig. 4). Sensitivity analyses (see “Methods”) show our results are robust to potential interspecific variation in morphological and physiological parameters.

### Climate change refugia for desert birds and PA coverage

**Fig. 2:**
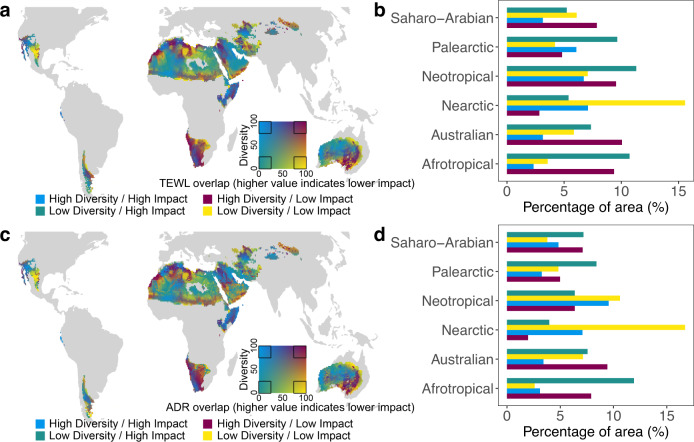
Overlapping projections of climate change impacts and the distribution of desert bird diversity. Climate change impacts are measured as the proportion of overlap between current and future values of TEWL (panels **a** and **b**) or ADR (panels **c** and **d**) per pixel (higher overlap implies lower impact) when global mean temperatures are 2 °C warmer than pre-industrial values. We defined desert bird as bird species with ≥90% area of their global habitat area falling within warm deserts. Diversity is calculated as rarity-weighted species richness, where species are weighted by the size of their global Area of Habitat (AOH). Panels **a** and **c** are bivariate heatmaps that place each pixel along axes of TEWL/ADR overlap and diversity value (from 0 to 100 percentiles; mapping is done for each desert realm). Correspondingly, panels **b** and **d** show the percentages of area in each desert realm falling within the four categories defined by TEWL/ADR overlap and diversity value (“High” and “Low” are defined by whether the pixel value falls in the top or bottom 25% of all pixel values within that desert realm, respectively). For each pixel, we averaged the results for birds in three body mass categories (see “Methods”), weighted by the number of bird species in each category, to calculate TEWL and ADR values. We assumed that a bird actively shifts between open and shaded habitats to minimize its rate of water loss. See Supplementary Fig. 9 for results assuming a bird always stays in the open. See Supplementary Figs. 10, 11 for results for a scenario in which global mean temperatures are 4 °C warmer than pre-industrial values.

We defined desert birds as species having ≥90% of their habitat within warm deserts. By overlaying the distribution of desert bird diversity (measured as rarity-weighted species richness^28^) with our projections of climate change impacts on desert birds, we generated bivariate heat maps for the world’s warm deserts that place each pixel along axes of the two variables (Fig. 2a, c). We then classified each pixel as falling into one of four categories, based on whether it falls in the top (“high”) or bottom (“low”) 25% of values for desert bird diversity and climate change impact (Fig. 2b, d).

The area that falls within each of the above categories varies from realm to realm based on the degree to which avian diversity and climate change impacts are spatially aligned. The choice of TEWL or ADR for estimating climate change impact changes the area of warm desert that falls into each category. For example, the percentage of desert area in the Neotropical desert realm that falls into the “High-Diversity/Low-Impact” category when using ADR to estimate climate change impact is about two-thirds of the value obtained when considering TEWL instead. This mismatch is due to the difference between the probabilistic distributions of climate change impact in each desert realm measured using TEWL and ADR.

**Fig. 3:**
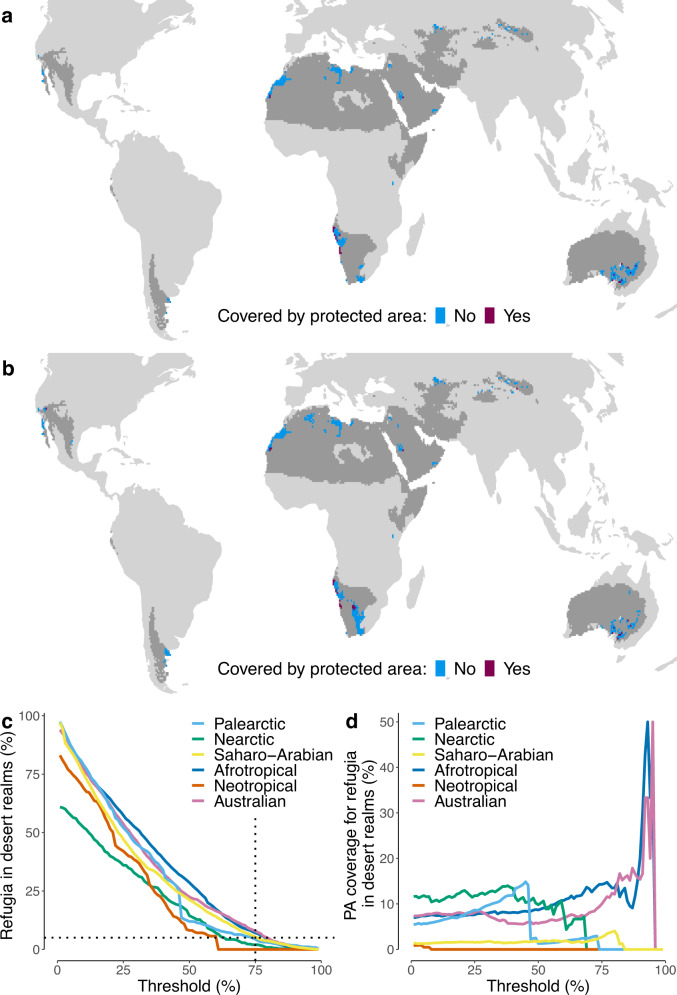
Predicted locations of climate change refugia for desert birds in global warm deserts and their current protection status. The figure considers a climate change scenario that the global mean temperatures are 2 °C warmer than pre-industrial values. Panel **a** shows the refugia identified using a fixed threshold of 75th percentile (i.e., top 25%) for ADR overlap, TEWL overlap, and avian diversity, while panel **b** shows the refugia identified using a floating threshold such that at least 5% of desert area in each realm is identified as refugia (see text for details). Panel **c** shows the relationship between the threshold used and the percentage of desert area in each realm identified as refugia (see “Source_data_Figure_3c” for source data). Panel **d** shows the relationship between the threshold used and PA coverage for refugia identified in each realm (see “Source_data_Figure_3d” for source data). We assumed that a bird actively shifts between open and shaded habitats to minimize its rate of water loss. See Supplementary Fig. 12 for results assuming a bird always stays in the open. See Supplementary Figs. 13, 14 for results for a scenario in which global mean temperatures are 4 °C warmer than pre-industrial values.

We defined refugia as areas in each desert realm with relatively high diversity and low climate change impact measured using either TEWL or ADR. We assumed that these three variables are equally important when considering biodiversity conservation, and therefore used the same threshold for all three to identify refugial areas. For example, a threshold of 75% means that we selected pixels in a given desert realm that had bird diversity values (larger values indicate higher diversity) and proportion of overlap in TEWL or ADR (larger values indicate lower impacts) larger than the 75th percentile of pixels for that realm. As the distributions of the three variables vary spatially within desert realms, using a fixed threshold for different desert realms could result in very different percentages of desert area being identified as refugia. Thus, we ran separate analyses in which we specified that a fixed percentage area of each desert realm must qualify as refugia, calculated by adjusting a “floating” threshold until that percentage area target was met. We did so under the assumption that every desert realm has unique value for biodiversity and therefore is worth protecting, notwithstanding differing impacts of climate change among realms. Results comparing the fixed and floating thresholds are shown in Fig. 3.

Using a fixed threshold of 75% for avian diversity and climate change impacts for all six desert realms (Fig. 3a), we found notable differences between desert realms with respect to the percentage area identified as refugia. The Australian desert realm has the largest percentage (6.6%) of its area identified as refugia, while the Neotropical desert realm has just 1.7% (see the vertical dotted line in Fig. 3c). Next, we adjusted these thresholds such that at least 5% of each desert realm’s area was identified as refugia (see Fig. 3b). The Australian, Afrotropical, and Saharo-Arabian desert realms maintain stricter thresholds (>75%) than the other three desert realms to meet the goal of 5% refugia (see the horizontal dotted line in Fig. 3c). To understand the extent to which these refugia are currently protected, we overlaid the boundaries of existing PAs with the identified refugia. The PA coverage for refugia in each desert realm is generally low (<20%), although percentages vary depending upon the threshold values used to define refugia (Fig. 3d).

### Comparing refugia identified using physiological metrics and those identified using Tair

Although our physiological modeling of climate change impacts (TEWL and ADR) on desert birds was positively correlated with those estimated using the overlap between current and future values of Tair, and negatively correlated with mean current Tair, the relative magnitude of impacts estimated using the these metrics was not spatially aligned (Weighted Jaccard Index ≤75.5%^29^; Supplementary Tables 4 and 5). The relative magnitudes of predicted climate change impacts between desert realms are contingent on whether or not physiological responses are considered. Also, the probability distribution of predicted climate change impacts in each desert realm is more positively skewed when considering TEWL or ADR than is the case for predicted impacts based solely on Tair. In other words, pixels showing very high climate change impact based on physiological variables are more extreme relative to all other pixels than is the case when considering only Tair (Supplementary Fig. 4).

Physiological modeling requires more than just climate data. A critical question, then, is whether refugia identified using the proportion of overlap between current and future values of Tair differ spatially from refugia identified using physiological metrics. If the use of Tair alone would result in both low under-protection (i.e., does not omit many of the refugia identified using physiological metrics, equivalent to false negatives) and low overprotection (i.e., does not identify as refugia extensive areas that are not identified as such using physiological metrics, equivalent to false positives), then Tair could provide a reasonably good proxy for the more appropriate but costlier physiological metrics. Unfortunately, when comparing refugia identified using Tair and those identified using physiology, we found considerable under-protection and over-protection in all desert realms (Supplementary Fig. 5). Under-protection and overprotection can both involve up to 60% of predicted refugia area in some desert realms.

## Discussion

Gaining a nuanced understanding of how climate change will affect species in the world’s warm deserts requires an integrated consideration of the spatial patterns of both biodiversity and physiological impacts. To better understand the distribution of physiological impacts to species due to climate change, we combined microclimate data, climate change projections, and physiologically explicit biophysical models to predict climate change impacts to birds across the world’s warm deserts for when global mean temperatures are 2 °C warmer than pre-industrial values. We found considerable heterogeneity of climate change impacts both between and within major warm deserts. Climate change refugia, areas with high desert bird diversity and low climate change impact, are predicted to differ markedly in total area between desert realms. Alarmingly, only a very small proportion of these refugia fall within the borders of existing PAs. Species within climate change refugia that occur outside PAs are exposed to potential harm from land-use change, overexploitation, and other direct human impacts^30,31^. Compared with projections based solely on air temperature, physiological models produced markedly different spatial patterns of climate change impacts on desert birds. Using air temperature as a proxy for physiological metrics results in under-protection of future refugia and overprotection of areas that are not expected to function as refugia. These conclusions hold even for high-risk scenarios wherein global mean temperatures are 4 °C warmer than pre-industrial levels or in which birds have no access to shade, extreme cases that indicate our findings are relatively robust.

Interestingly, no matter which methods are used, most identified refugia lie close to coastlines, which may be related to the oceanic buffering effect for terrestrial warming^32^. However, sea-level rise^33^ and increasing human disturbance^34^ may reduce available habitats in these areas. In the context of recent calls to increase global land protection to 30% by 2030^35^, we propose that refugia we have identified be considered in future conservation efforts as a way to ensure that desert species persist in the face of climate change. Of the six desert realms, the Neotropical desert realm has the lowest proportion of its predicted refugia currently within the boundaries of PAs. We note that our focus here is protecting sites that are likely to retain the greatest richness of desert birds in the future. Models for other taxonomic groups should be developed to determine whether their refugia overlap significantly with the avian refugia we have identified. We emphasize that our results in no way imply that desert areas falling outside the boundaries of refugia are unworthy of conservation attention. For example, an additional reasonable objective would be to reduce harmful land-use changes in high diversity areas that are predicted to suffer greatly from climate change to minimize additional anthropogenic stressors to the species living there. Nor do we wish to imply that our results represent the “final word” as to which places will function as climate refugia for birds. Future refinements to the models we used can enhance their predictive value.

Finally, our study highlights the value of using physiologically explicit biophysical models parameterized with microclimate data to predict how organisms will actually experience climate change, and it provides a physiologically relevant framework for prioritizing desert areas for future protection.

## Methods

### Global warm deserts and terrestrial zoogeographic realms

We created a map of global warm deserts (with a resolution of 50 km) by choosing desert-related habitat types from a global map of terrestrial habitat types^36^, based on the Habitat Classification Scheme of IUCN (version 3.1). The habitat types we chose included: hot desert, temperate desert, subtropical/tropical dry shrubland, subtropical/tropical dry lowland grassland, and dry savanna. We further refined this map by restricting it to areas with less than 500 mm of annual precipitation (using averaged data for 1970–2000; WorldClim V2.1^37^), which is a widely-used threshold for identifying arid or semi-arid regions^38^. We divided global warm deserts into six major realms (desert realms) using an updated map of Wallace’s zoogeographic regions of the world^39^.

### Climate data and microclimate model

We used historical and projected future monthly climate data from TerraClimate^40^ (50 km spatial resolution) for our simulations, which provides maximum temperature, minimum temperature, precipitation, soil moisture, vapor pressure, downward surface shortwave radiation, and wind-speed. Two future climate scenarios were considered: (1) when global mean temperatures are 2 °C warmer than pre-industrial values, and (2) when global mean temperatures are 4 °C above pre-industrial values. The climate change scenarios were derived from 23 CMIP5 global climate models and downscaled using a pattern-scaling approach described in Qin et al. 2020^41^. The ‘micro_terra’ function of NicheMapR then disaggregated the monthly climate data to hourly following methods described in Kearney and Porter (2017)^42^. Using this function, temperature data are elevation- and terrain-corrected, spline interpolated to daily, and then downscaled to hourly by imposing a latitude-and longitude-dependent diurnal cycle to the data. Hourly relative humidity is then determined from the vapor pressure and modeled diurnal variation in air temperature. Radiation is interpolated to hourly, by computing the clear sky fraction in each month and then computing hourly clear sky radiation. See Supplementary Table 1 for parameter values for the microclimate model. We extracted monthly climate data for global warm deserts for what is typically the hottest month (July and January for Northern and Southern Hemispheres, respectively).

### Model species and physiological model

Desert birds exhibit diversity in their morphology, behavior, and physiology^43–45^, which may affect their sensitivity to climate change. Except for body size and morphological traits that scale with body size, empirical data for many traits are available only for a few species, which precludes us from running the physiological model for every single desert species. However, previous studies have suggested that body size significantly affects TEWL^26^ and ADR^13^, so we created three model species weighing 13 g, 39 g, and 185 g, representing small (0–33th percentiles), medium (33–66th percentiles) and large (66–100th percentiles) desert birds (Supplementary Data 1). Specifically, we first created the medium-size model species by using size-related traits (body mass, plumage depths, feather lengths) of the desert-dwelling Cactus Wren (*Campylorhynchus brunneicapillus*; body mass 39 g). Other parameters were taken either from the Cactus Wren and other well-studied species or were based on our best estimates (Supplementary Data 2). We then created the small-size and large-size model species by adjusting size-related traits (plumage depths and feather lengths, which were scaled to the mass to the power of 1/3). Model results generated using the three model species were averaged for each desert grid cell weighted by the number of species falling within each size category.

We calculated the hourly water loss (cutaneous water loss + respiratory water loss) of bird species using a customized version of the “endoR_devel” function in R package “NicheMapR”^46^. This function implements a biophysical model for calculating heat and mass exchange between an individual endotherm and a given environment, and for simulating required postural and physiological thermoregulation for maintaining minimal metabolic rates. Our model found a solution for maintaining minimal metabolic rates at all desert sites. Based on the physiology of desert birds^44,45^, we revised the sequence of thermoregulatory events for all our model species in the face of heat stress as follows: (1) reduce ptiloerection; (2) stretch the body; (3) increase flesh conductivity; (4) simultaneously raise core temperature (up to 44 °C) and respiratory rate (up to 7.5 times of the resting level), by modifying the source code of “endoR_devel”. We also converted and run the function with Fortran for a faster running speed. We assumed that birds sit 1.5 m above ground and shifted between open (0% shade) and shady areas (90% shade) to minimize their hourly water loss. As deep shade may not be widely available in deserts and shade-seeking behavior may involve trade-offs with other behaviors such as foraging^47^, we considered a scenario in which the bird always stayed in an open area.

We calculated the TEWL as total water loss in an average day of the typically hottest month of the year^20,26^ and the ADR as the maximum water loss per gram of mass in three continuous hours in an average day of the typically hottest month of the year. The ADR reflects the risk of the bird dying due to acute dehydration as previous studies have suggested that birds are unlikely to survive when accumulated water loss reaches 15% of body mass within three hours^13,19,27^. We projected maps of air temperature and physiological results using the Eckert IV equal-area projection to ensure each pixel represents the same area.

### Model validations

We validated model predictions of body temperature and water loss rate at a series of air temperatures against empirical data for well-studied species from four orders and nine families (only size-related traits were adjusted for each species based on body mass). The results indicate a good performance of our physiological models (see Supplementary Figs. 1, 2). We collected empirical data of core body temperature and evaporative water loss rate measured at air temperatures using a flow-through respirometry system from literature^44,45,48,49^. In total, data of nine bird species from four orders and nine families were collected. We predicted the core body temperature and evaporative water loss at air temperatures using the physiological model (customized “endoR_devel” from NicheMapR) and parameters that we used for the main analysis. To simulate the experimental condition, we used a wind speed of 0.1 m/s, a relative humidity of 5% and zero radiation. We adjusted only five size-related parameters to account for size variation among species: body mass, plumage depth (dorsal and ventral) and feather length (dorsal and ventral), which is the same method we used in the main analysis. The body mass (AMASS) of species were from the literature and we scaled plumage depths and feather lengths (known for *Campylorhynchus brunneicapillus*) in proportion to AMASS^1/3^^50,51^. The results suggested that our model predicts the core body temperature and evaporative water loss of bird species well (Supplementary Figs. 1, 2). Note that our model slightly overestimated the body temperature and water loss rate of some non-passerine species, which was because we used a passerine metabolic rate equation for all species. We decided to use one equation for basal metabolic rate (from McNab et al. 2009 for passerine) for all desert birds for the following reasons: 1. Additional sensitivity analysis suggested that using the QBASAL equation for passerines or non-passerines does not largely change identified safe sites (see Supplementary Tables 2, 3); 2. Over 77% (118 out of 152) desert bird species (bird species having $\ge$ 90% of their habitat within warm deserts) are passerines; 3. Some non-passerine species show high metabolic rates, while some passerine species show low metabolic rates^52^.

We validated our model predictions of changes in TEWL and ADR from the period of 1911–1940 to the period 1971–2000 against observed occupancy declines of bird species in the Mojave Desert^26^. The results indicate that the changes in TEWL and ADR predicted by our model were negatively correlated with the changes in occupancy (Supplementary Fig. 3; *p* < 0.05). We collected the changes in occupancy of 50 bird species in the Mojave Desert over the past century from Riddell et al. (2019)^26^. We predicted the changes in average TEWL in July (TEWL; g/day) from the period of 1911–1940 and 1971–2000 at a representative site (Mojave Desert National Preserve [35°00′39″ N, −115°28′24″ W]), which represent the climate in the Mojave Desert of the two survey periods. The climate data for the two periods were from the California Basin Characterization Model dataset^53^. We used the same microclimate model and physiological model we used in the main analysis for the model prediction. We adjusted only five size-related parameters to account for size variation among species: body mass, plumage depths (dorsal and ventral), and feather lengths (dorsal and ventral). The body mass (AMASS) of species were from Riddell et al. (2019)^26^ and we scaled plumage depths and feather lengths in proportion to AMASS^1/3^^50,51^. The result suggested that our model predictions of changes in TEWL (*p* = 0.028) and ADR (*p* = 0.031) were negatively correlated with the change in occupancy (linear model used; Supplementary Fig. 3).

### Sensitivity analysis

We conducted sensitivity analyses to test if conclusions generated from our models were robust to interspecific variation in species traits in ways that might affect the water loss rates. As the aim of this study is to identify the areas within deserts that are safer for birds under climate change relative to other desert areas, provided the variation in species traits does not affect the relative rankings of these desert areas, we can rely on results generated using the model species to identify climate change refugia. Therefore, we performed a sensitivity analysis by rerunning the model using much lower or higher (but nonetheless realistic) parameter values for desert birds and then identifying “safe sites” (defined in each case as the top 25% of sites showing the largest overlap between current and future values of TEWL and ADR). We then noted the sites that consistently appear in this top quarter. The results of this analysis indicate that our conclusions are robust to potential interspecific variation in traits (Supplementary Tables 2, 3).

We ran sensitivity analyses by calculating the overlap between current (1986–2015) and future (pseudo years 1986–2015 commensurate with the climate future that the global mean temperatures are 2 °C warmer than pre-industrial values) values of the total evaporative water loss (TEWL; g/day) and acute dehydration risk (ADR; % mass) in the hottest month at desert sites in Australia (1627 sites) using different parameter values. We tested the sensitivity of identified 25% sites with the largest TEWL overlap or the largest ADR overlap (safe sites) between the two time periods to potential interspecific variations in model parameters (Supplementary Tables 4 and 5). For each model parameter, we reran the models using extreme parameter values that were lower and higher (but realistic for desert birds) than the values we used for the model species, while keeping other parameters unchanged. For the assumed sitting height, we conducted the sensitivity analysis using a lower value considering that some desert birds only use terrestrial habitats (e.g., Otidiformes). For basal heat generation, we used a function of body mass for non-passerine species in the sensitivity analysis. For the assumed onset of panting, we conducted the sensitivity analysis for a scenario that the bird starts panting after reaching its maximum body temperature. We also conducted sensitivity analyses for combinations of parameters set at extreme values that would minimize and maximize the water loss rate. The results suggested that at least 69.3% (over 90% in most cases) of identified safe sites using the model species can be consistently predicted using lower or higher parameter values or combinations of parameter values that maximize or minimize the water loss rate.

### Rarity-weighted richness and PAs

The maps of the spatial distribution of bird species were downloaded from BirdLife^54^ and refined to Area of Habitat (AOH)^55^, based on species-specific habitat and elevation requirements listed by BirdLife. We defined desert bird species as bird species with more than 90% of their AOH falling within warm deserts (152 species). We then calculated the rarity-weighted species richness (RWR) of desert bird species for each grid cell by summing the inverse of each species’ AOH for all species occurring in that cell (following Kier et al. 2009^28^). RWR (sometimes called endemism richness) better captures the relative importance of an area for global biodiversity than does unweighted species richness (which can be dominated by common, widespread species), by assigning higher values to species with smaller ranges, therefore incorporating aspects of both richness and endemism^28^.

We calculated the overlapped estimated area of kernel density estimations (using the “overlap” function in R package “overlapping”^56^) for current and future values of TEWL and ADR for three modeled birds with small, medium, and large body masses. To compare rarity-weighted richness with these physiological responses under climate change scenarios, we calculated the average predicted TEWL overlap and ADR overlap for the avian community that occurs in each location, weighted by the number of species in each of the three body mass categories.

To assess PA coverage we overlaid refugia with a global map of PAs provided by the World Database on Protected Areas (WDPA)^57^, refined following Butchart et al. 2015^58^. We considered only strictly PAs in the categories “Ia”, “Ib”, “II”, “III” and “IV”, as defined by the IUCN Protected Area Categories System^59^.

### Statistical analysis

We used Kruskal–Wallis tests to compare the changes in and overlap between the distributions of current and future values of Tair, TEWL and ADR between desert realms, and used Epsilon-Squared (R package “rcompanion”^60^) as the corresponding effect size statistic. We used pairwise Pearson correlation tests to estimate correlations between projected climate change impacts based on TEWL, ADR, and Tair, respectively. To compare the spatial similarity between maps of projected climate change impacts, we used a weighted version of the Jaccard similarity index^29^. The Jaccard index is a measure of the proportion of shared elements between two maps, and the weighted version allows for the comparison of two maps with values along a continuous gradient^61^. We compared maps for the predicted overlap between current and future values in TEWL, ADR, and Tair in pairs. We also used above methods to compare the climate-change impacts estimated using TEWL and ADR with mean current air temperature.

### Softhware

Maps of AOH, warm deserts and rarity-weighted richness were created using Google Earth Engine^62^. All other analyses were performed in R 4.0.3^63^.

### Inclusion & ethics

Our research has included researchers from countries around the world that contain warm deserts. Roles and responsibilities were agreed amongst collaborators ahead of the research. We have taken local and regional research relevant to our study into account in citations.

### Reporting summary

Further information on research design is available in the Nature Portfolio Reporting Summary linked to this article.

## Supplementary information

Supplementary Information

Description of Additional Supplementary Information

Supplementary Data 1

Supplementary Data 2

Supplementary Data 3

Supplementary Data 4

Supplementary Data 5

Reporting Summary

## Data Availability

Habitat classification scheme of IUCN (version 3.1): https://github.com/Martin-Jung/Habitatmapping; WorldClim 2: http://www.worldclim.com/version2; An updated map of Wallace’s zoogeographic regions of the world: https://macroecology.ku.dk/resources/wallace; TerraClimate: https://www.climatologylab.org/terraclimate.html; Maps of the spatial distribution of bird species from BirdLife: http://datazone.birdlife.org/species/requestdis; World Database on Protected Areas (WDPA): www.protectedplanet.net; California Basin Characterization Model dataset: http://climate.calcommons.org/bcm. Source data are provided for Fig. 3c (Supplementary Data 3) and Fig. 3d (Supplementary Data 4). Climate change impact data, bird diversity data, protected area coverage data generated in this study are provided as Supplementary Data 5.

## Code availability

Codes used in this analysis have been deposited in Zenodo: 10.5281/zenodo.7088572
