# Author Correction: Global patterns of climate change impacts on desert bird communities

**DOI:** 10.1038/s41467-023-36191-y

**Published:** 2023-01-25

**Authors:** Liang Ma, Shannon R. Conradie, Christopher L. Crawford, Alexandra S. Gardner, Michael R. Kearney, Ilya M. D. Maclean, Andrew E. McKechnie, Chun-Rong Mi, Rebecca A. Senior, David S. Wilcove

**Affiliations:** 1 Princeton School of Public and International Affairs, Princeton University, Princeton, NJ USA; 2 School of Ecology, Shenzhen Campus of SunYat-sen University, Shenzhen, Guangdong People’s Republic of China; 3 South African Research Chair in Conservation Physiology, South African National Biodiversity Institute, 2 Cussonia Ave, Brummeria, Pretoria 0184 South Africa; 4 DSI-NRF Centre of Excellence at the FitzPatrick Institute, Department of Zoology and Entomology, University of Pretoria, Lynnwood Rd., Pretoria, 0002 South Africa; 5 Environment and Sustainability Institute, University of Exeter Penryn Campus, Penryn, Cornwall TR10 9FE UK; 6 School of BioSciences, The University of Melbourne, Melbourne, VIC 3010 Australia; 7 Key Laboratory of Animal Ecology and Conservation Biology, Institute of Zoology, Chinese Academy of Sciences, Beijing, People’s Republic of China; 8 Conservation Ecology Group, Department of Biosciences, Durham University, Durham, DH1 3LE UK; 9 Department of Ecology and Evolutionary Biology, Princeton University, Princeton, NJ USA

**Keywords:** Conservation biology, Climate-change ecology, Biodiversity, Ecological modelling

Correction to: *Nature Communications* 10.1038/s41467-023-35814-8, published online 13 January 2023

The original version of this Article contained an error in the author affiliations. The affiliation of Rebecca Senior with Conservation Ecology Group, Department of Biosciences, Durham University, Durham DH1 3LE, UK was inadvertently omitted. This has now been corrected in both the PDF and HTML versions of the article.

