# Supplementary material for: Global patterns of climate change impacts on desert bird communities
Source: Nat Commun. 2023 Jan 13;14:211. doi: 10.1038/s41467-023-35814-8 (PMC9839677; doi:10.1038/s41467-023-35814-8)
Supplement: Supplementary file 1 — Supplementary Information [file 41467_2023_35814_MOESM1_ESM.pdf]

Global Patterns of Climate Change Impacts on Desert Bird Communities

Supplementary Tables

Supplementary Table 1 Parameter values for the microclimate model ("micro_terra" in NicheMapR) that define the desert habitat.

Parameter	Description	Value	Comment
REFL	Soil solar reflectance, decimal %	0.35	table 11.2 in Campbell and Norman (2012) [1]
Usrhyt	Local height (m) at which air temperature, wind speed and humidity are to be computed for organism of interest	1.5	assumed that birds were sitting 1.5 m above ground
RUF	Roughness height (m), e.g. smooth desert is 0.0003, closely mowed grass may be 0.001, bare tilled soil 0.002-0.006, current allowed range: 0.00001 (snow) - 0.02 m.	0.0003	best estimate for deserts
CMH2O	Precipitable cm H2O in air column, 0.1 = very dry; 1.0 = moist air conditions; 2.0 = humid, tropical conditions (note this is for the whole atmospheric profile, not just near the ground)	0.5	best estimate for deserts

Supplementary Table 2 Parameter values used in the sensitivity analysis and percentage of safe sites that were defined as the 25% sites with the largest TEWL overlap between current years and future years (global mean temperatures are 2°C warmer than pre-industrial values) that were predicted.

Parameter	Description	Value used for the model species	Lower value used and percent of safe sites predicted	Higher value used and percent of safe sites predicted
REFLD	feather reflectivity dorsal (fractional, 0-1)	0.25	0.1; 98.0%	0.5; 97.8%
REFLV	feather reflectivity ventral (fractional, 0-1)	0.43	0.1; 97.5%	0.5; 98.5%
TC_MAX	maximum core temperature (°C)	44	43; 97.8%	45; 98.0%
PANT_MULT	multiplier on basal metabolic rate at maximum panting level	1.01	1; 98.3%	2; 86.5%
PANT_MAX	maximum breathing rate multiplier to simulate panting	7.5	4; 97.0%	11; 95.5%
Usrhyt	Local height (m) at which air temperature, wind speed and humidity are to be computed for organism of interest (m)	1.5	0.3; 95.3%	NA
			**Different equation or assumption and percent of safe sites predicted**	
QBASAL	basal heat generation (W)	0.278*0.13*mass^0.713	0.278*0.089*mass^0.724; 86%	
Onset of panting		Start with the increase of body temperature	Start after reaching maximum body temperature; 95.5%	
			**Percent of safe sites predicted**	
A combination of above parameter values that maximize the water loss rate (values in red)		NA	87.3%	
A combination of above parameter values that minimize the water loss rate (values in blue)		NA	83.0%	

Supplementary Table 3 Parameter values used in the sensitivity analysis and percentage of safe sites that were defined as the 25% sites with the largest ADR overlap between current years and future years (global mean temperatures are 2°C warmer than pre-industrial values) that were predicted.

**Parameter**	**Description**	**Value used for the model species**	**Lower value used and percent of safe sites predicted**	**Higher value used and percent of safe sites predicted**
REFLD	feather reflectivity dorsal (fractional, 0-1)	0.25	0.1; 94.5%	0.5; 91.0%
REFLV	feather reflectivity ventral (fractional, 0-1)	0.43	0.1; 91.8%	0.5; 95.3%
TC_MAX	maximum core temperature (°C)	44	43; 91.3%	45; 91.3%
PANT_MULT	multiplier on basal metabolic rate at maximum panting level	1.01	1; 94.5%	2; 88.0%
PANT_MAX	maximum breathing rate multiplier to simulate panting	7.5	4; 88.5%	11; 87.3%
Usrhyt	Local height (m) at which air temperature, wind speed and humidity are to be computed for organism of interest (m)	1.5	0.3; 87.8%	NA
			**Different equation or assumption and percent of safe sites predicted**	
QBASAL	basal heat generation (W)	0.278*0.13*mass^0.713	0.278*0.089*mass^0.724; 82.3%	
Onset of panting		Start with the increase of body temperature	Start after reaching maximum body temperature; 88.0%	
			**Percent of safe sites predicted**	
A combination of above parameter values that maximize the water loss rate (values in red)		NA	77.3%	
A combination of above parameter values that minimize the water loss rate (values in blue)		NA	69.3%	

Supplementary Table 4 Pearson Correlation Coefficient (estimated using a two-sided Pearson Correlation test) and Weighted Jaccard Similarity for pairwise comparison between metrics (Tair, TEWL and ADR) for estimating the climate-change impacts on global warm deserts. Climate change impacts were estimated by overlapping estimated area of kernel density estimations of current (1986-2015) and future values of Tair, TEWL and ADR, respectively. We considered two behavior assumptions (the bird shifting between open and shady areas to minimize their hourly water loss or always staying in the open) and two climate change scenarios (global mean temperatures being 2°C or 4°C warmer than pre-industrial values).

Result A	Result B	Behavior	Scenario	Correlation Coefficient	*P* value	Weighted Jaccard Index
Tair overlap	TEWL overlap	Shift	+2°C	0.818	0	68.6%
Tair overlap	ADR overlap	Shift	+2°C	0.799	0	75.5%
TEWL overlap	ADR overlap	Shift	+2°C	0.868	0	75.1%
Tair overlap	TEWL overlap	Open	+2°C	0.753	0	72.4%
Tair overlap	ADR overlap	Open	+2°C	0.657	0	72.1%
TEWL overlap	ADR overlap	Open	+2°C	0.886	0	78.1%
Tair overlap	TEWL overlap	Shift	+4°C	0.699	0	56.6%
Tair overlap	ADR overlap	Shift	+4°C	0.569	0	50.9%
TEWL overlap	ADR overlap	Shift	+4°C	0.867	0	62.8%
Tair overlap	TEWL overlap	Open	+4°C	0.863	0	48.9%
Tair overlap	ADR overlap	Open	+4°C	0.782	0	40.8%
TEWL overlap	ADR overlap	Open	+4°C	0.870	0	67.8%

Supplementary Table 5 Pearson Correlation Coefficient (estimated using a two-sided Pearson Correlation test) and Weighted Jaccard Similarity for pairwise comparison between physiological metrics (overlap between current and future values of TEWL / ADR) for estimating the climate-change impacts and mean current air temperature. Climate change impacts were estimated by overlapping estimated area of kernel density estimations of current (1986-2015) and future values of TEWL and ADR, respectively. We considered two behavior assumptions (the bird shifting between open and shady areas to minimize their hourly water loss or always staying in the open) and two climate change scenarios (global mean temperatures being 2°C or 4°C warmer than pre-industrial values).

Result A	Result B	Behavior	Scenario	Correlation Coefficient	*P* value	Weighted Jaccard Index
Tair	TEWL overlap	Shift	+2°C	-0.468	0	36.8%
Tair	ADR overlap	Shift	+2°C	-0.378	8.488×10^{-271}	46.6%
Tair	TEWL overlap	Open	+2°C	-0.454	0	43.0%
Tair	ADR overlap	Open	+2°C	-0.427	0	51.4%
Tair	TEWL overlap	Shift	+4°C	-0.311	7.053×10^{-181}	10.5%
Tair	ADR overlap	Shift	+4°C	-0.248	2.789×10^{-113}	15.2%
Tair	TEWL overlap	Open	+4°C	-0.282	2.770×10^{-147}	17.6%
Tair	ADR overlap	Open	+4°C	-0.241	7.481×10^{-107}	16.5%

Supplementary Figures

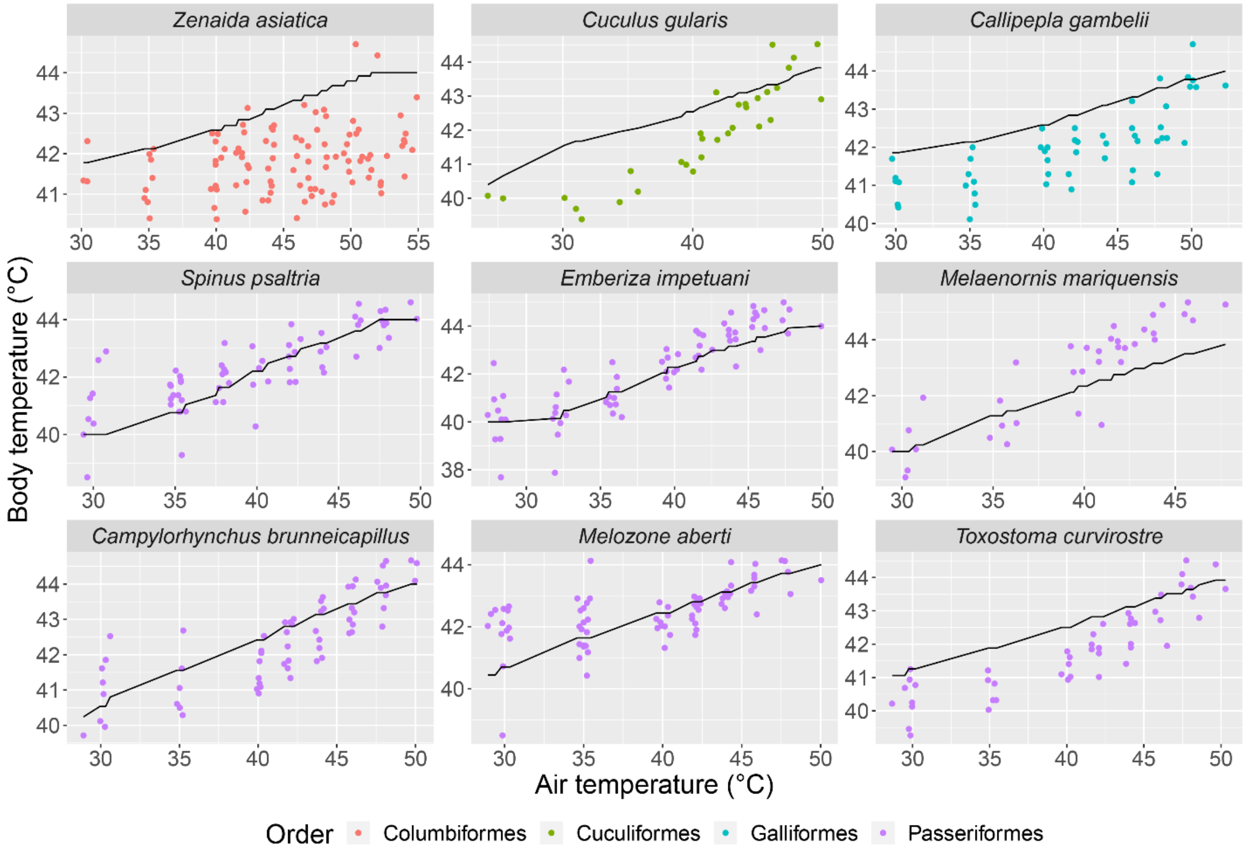

Supplementary Figure 1 Core body temperature at a series of air temperatures in nine bird species from four orders and nine families (*Z. asiatica*, *C. gambelii*, *C. brunneicapillus* and *T. curvirostre* use desert habitat according to IUCN Red List). The points represent empirical values measured using a flow-through respirometry system (from literature) and the lines represent corresponding predicted values generated by the physiological model.

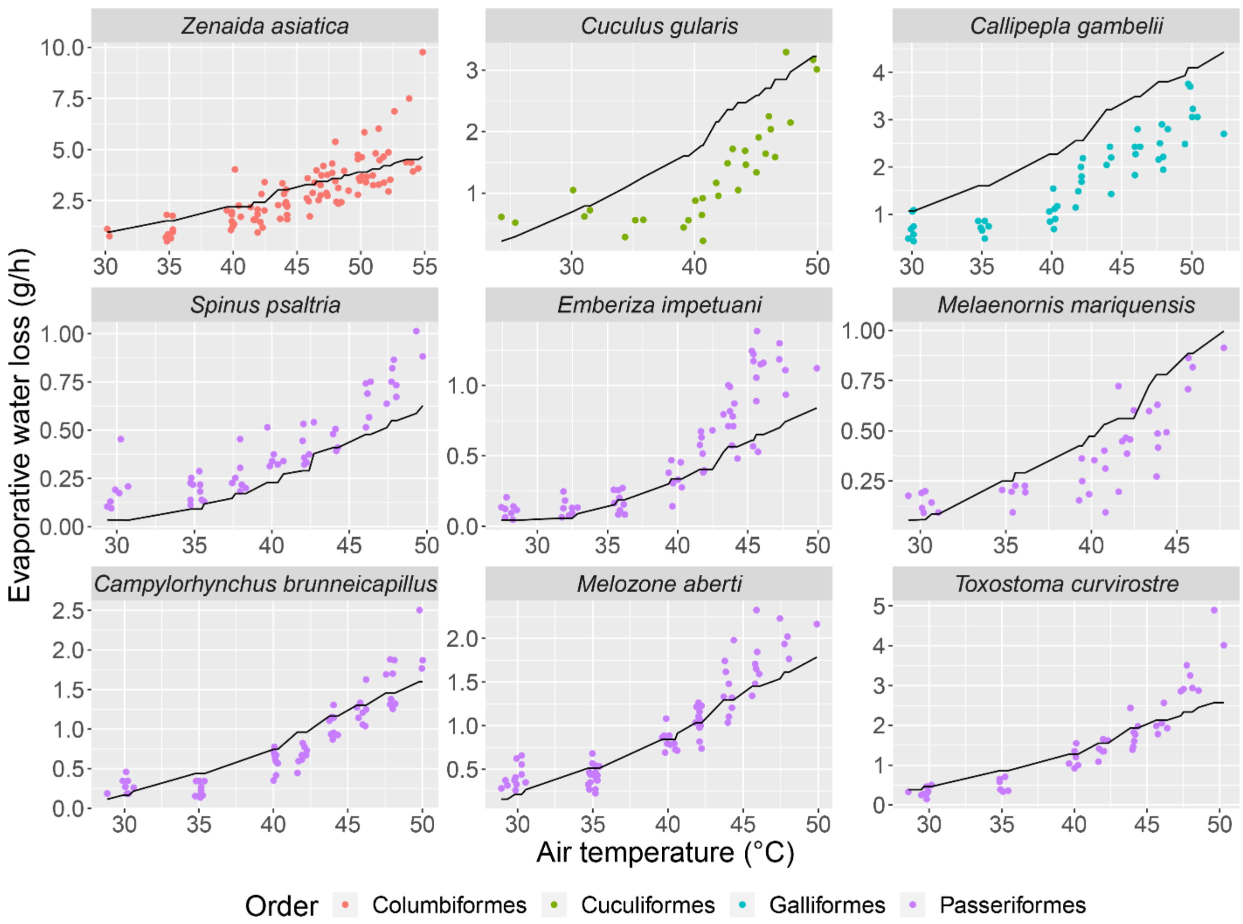

Supplementary Figure 2 Evaporative water loss at a series of air temperatures in nine bird species from four orders and nine families (*Z. asiatica*, *C. gambelii*, *C. brunneicapillus* and *T. curvirostre* use desert habitat according to IUCN Red List). The points represent empirical values measured using a flow-through respirometry system (from literature) and the lines represent corresponding predicted values generated by the physiological model.

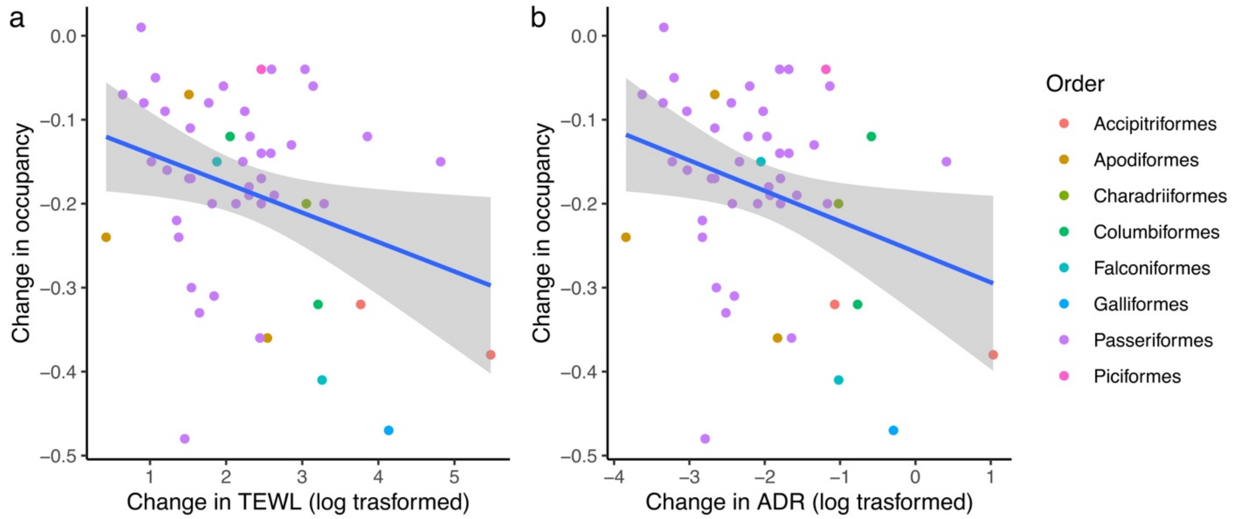

Supplementary Figure 3 Predicted change in total evaporative water loss (g/day) of 50 bird species in the Mojave Desert and their changes in occupancy over the past century. The shaded areas around fitted lines represent the 95% confidence intervals.

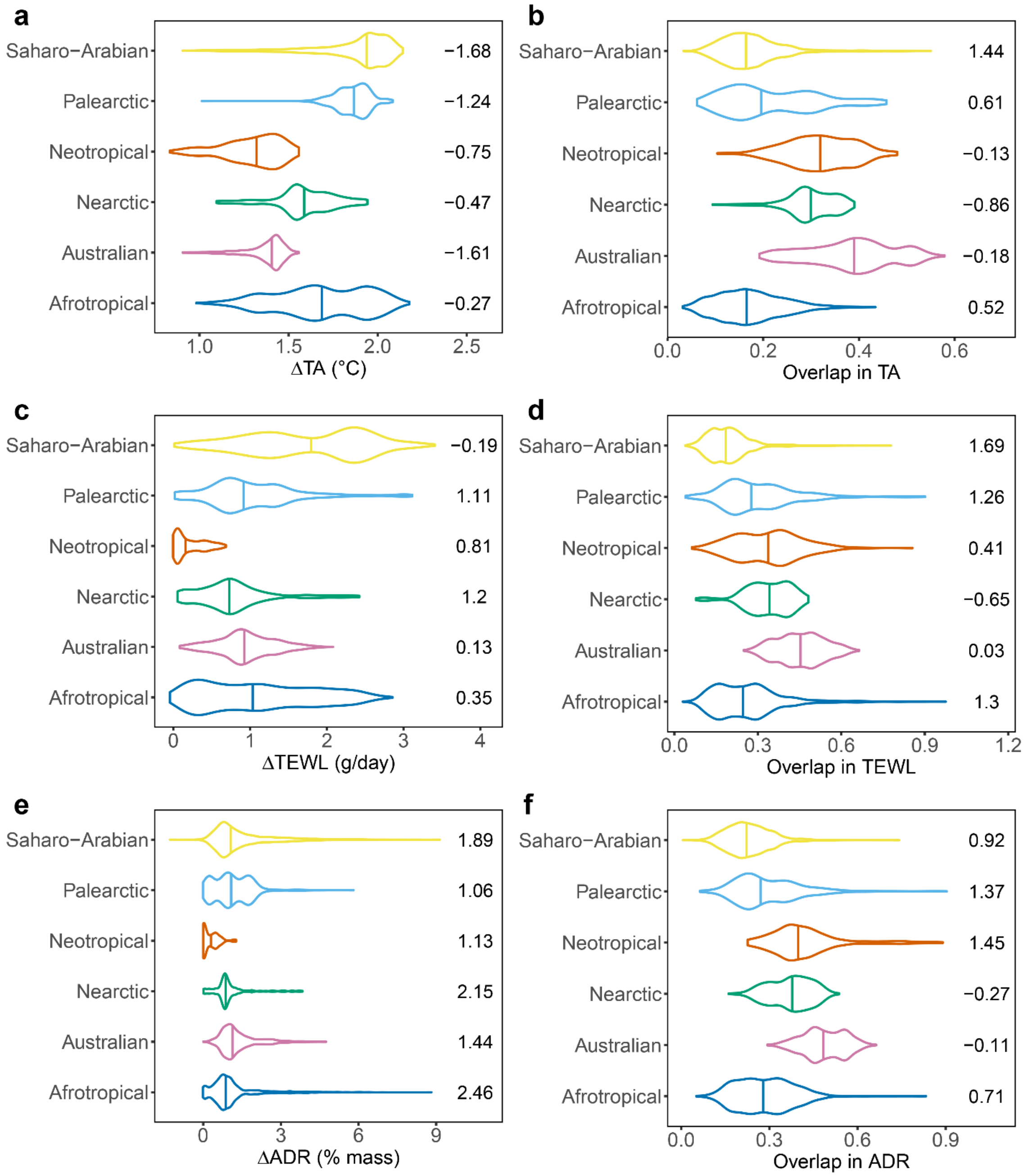

Supplementary Figure 4 Climate change impacts when global mean temperatures are **2°C** warmer than pre-industrial values. The climate change impacts are estimated as the changes in (a, c and e; "Δ" represent value changes) and the overlap (b, d and f) between current and future values of mean air temperature (Tair; °C), total evaporative water loss (TEWL; g/day) and acute dehydration risk (ADR; %mass) during the hottest month (July for Northern Hemisphere, January for Southern Hemisphere) in global warm deserts. The violin plots show the probability distributions of realm-based values in corresponding to those metrics. The vertical line in each violin plot indicates the 50th percentile of the distribution. The numbers on the right of the violin plots indicate the skewness of the distribution (more positive values indicate positive [i.e., right] skewness). This figure assumed that the bird **actively shifts between open and shaded habitats** to minimize its water loss rate.

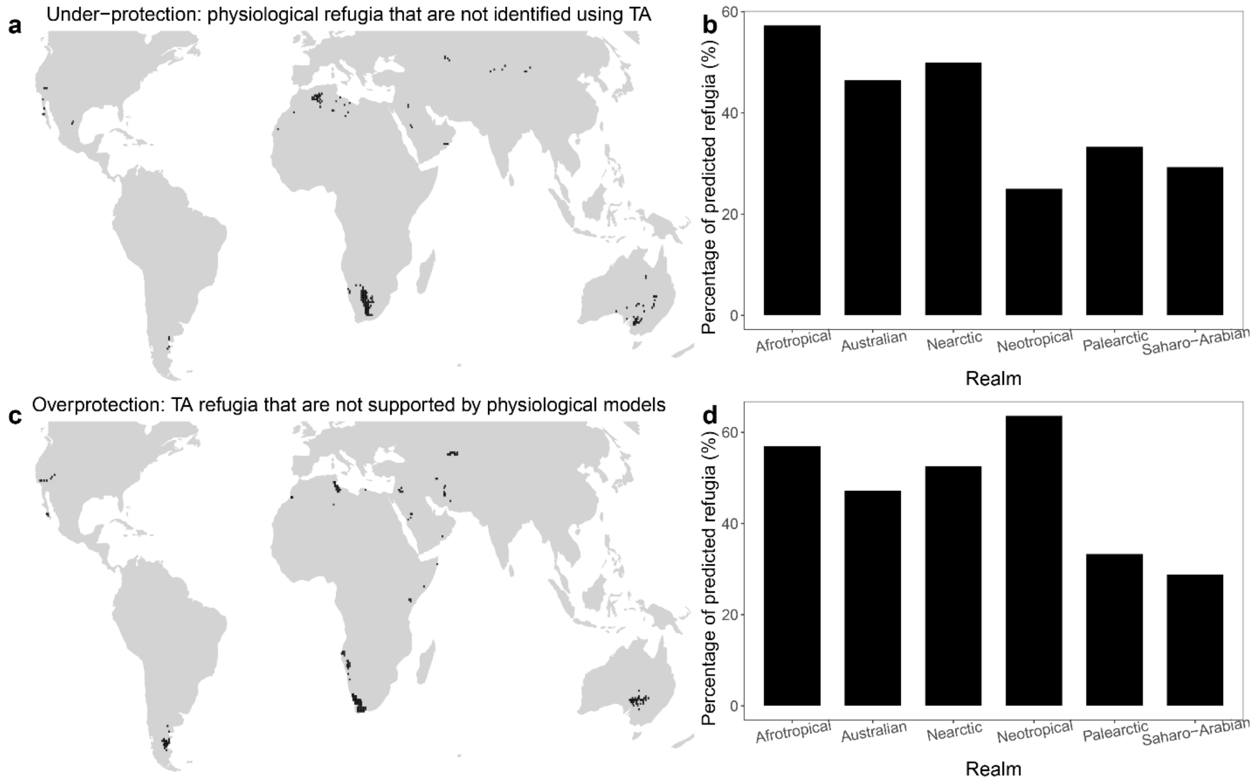

Supplementary Figure 5 Under-protection and overprotection when using air temperature as a proxy for physiological metric in identifying climate change refugia. The figure considers a climate change scenario that the global mean temperatures are **2°C** warmer than pre-industrial values. Panel a shows areas identified as refugia using physiological metrics that are not identified as refugia using Tair (i.e., “under-protection”); panel c shows areas identified as refugia using Tair that are not identified as refugia using physiological metrics (i.e., “overprotection”). Panel b and d show percentage of desert area in each desert realm where the “under-protection” and “overprotection” occurs, respectively. This figure assumed that the bird actively **shifts between open and shaded habitats** to minimize its water loss rate. See Fig. S15 for results assuming the bird always stays at open habitat. See Fig. S16-17 for results for a scenario that the global mean temperatures are 4°C warmer than pre-industrial values.

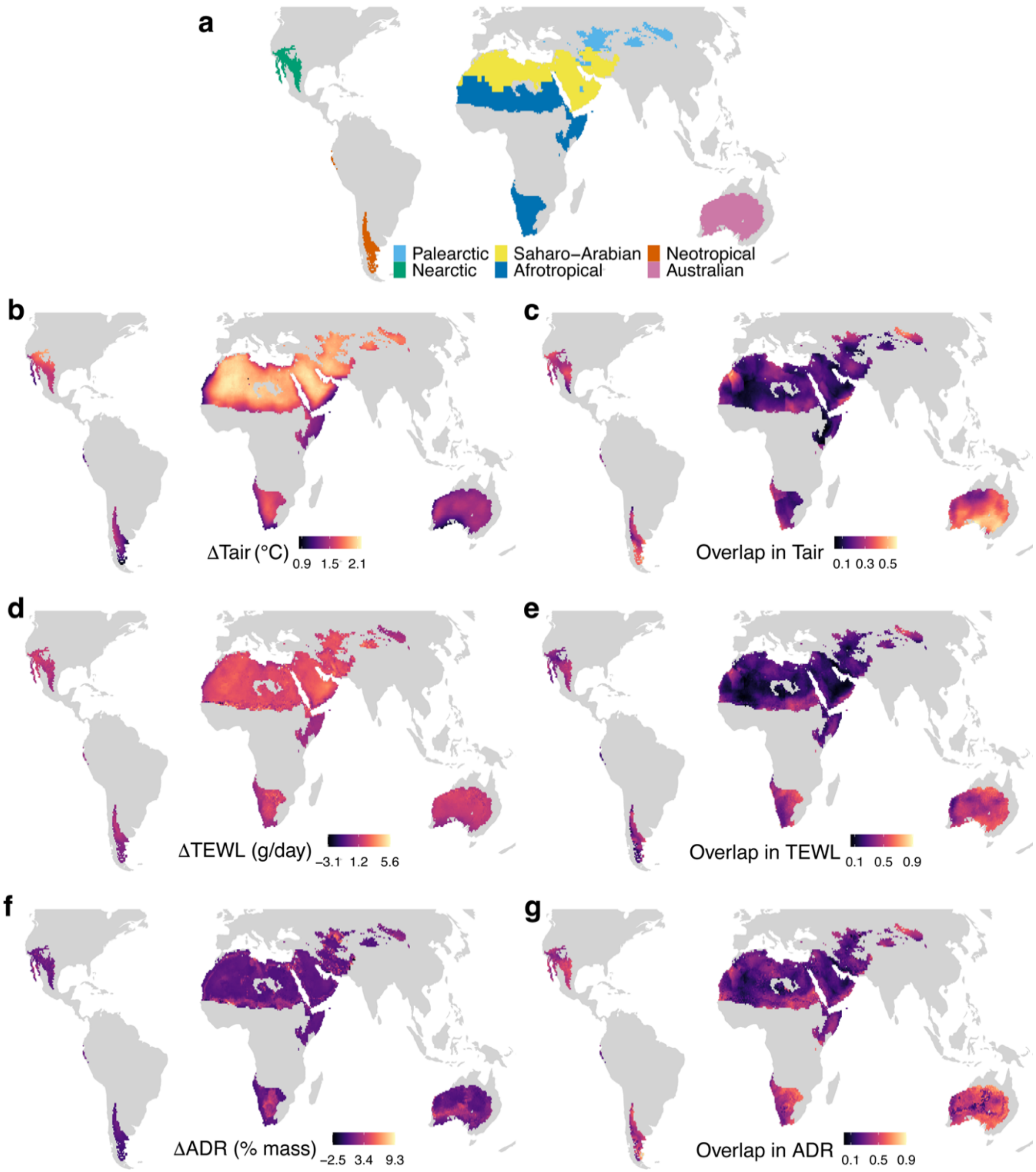

Supplementary Figure 6 Climate change impacts for desert birds when global mean temperatures are **2°C** warmer than pre-industrial values. The climate change impacts are shown as estimated changes in mean values (panels b, d, and f; "Δ" represent value changes; warmer colors indicate higher impact) and proportion of overlap between current and future values (panels c, e, and g; cooler colors indicate higher impact) of air temperature (Tair; °C), total evaporative water loss (TEWL; g/day) and acute dehydration risk (ADR; percent of body mass) during the hottest month (July for Northern Hemisphere, January for Southern Hemisphere). Panel a shows the locations of the six major realms containing warm deserts ("desert realms") and desert birds (bird species having ≥ 90% of their habitat within warm deserts). This figure assumed that the bird always stays in **open habitat**.

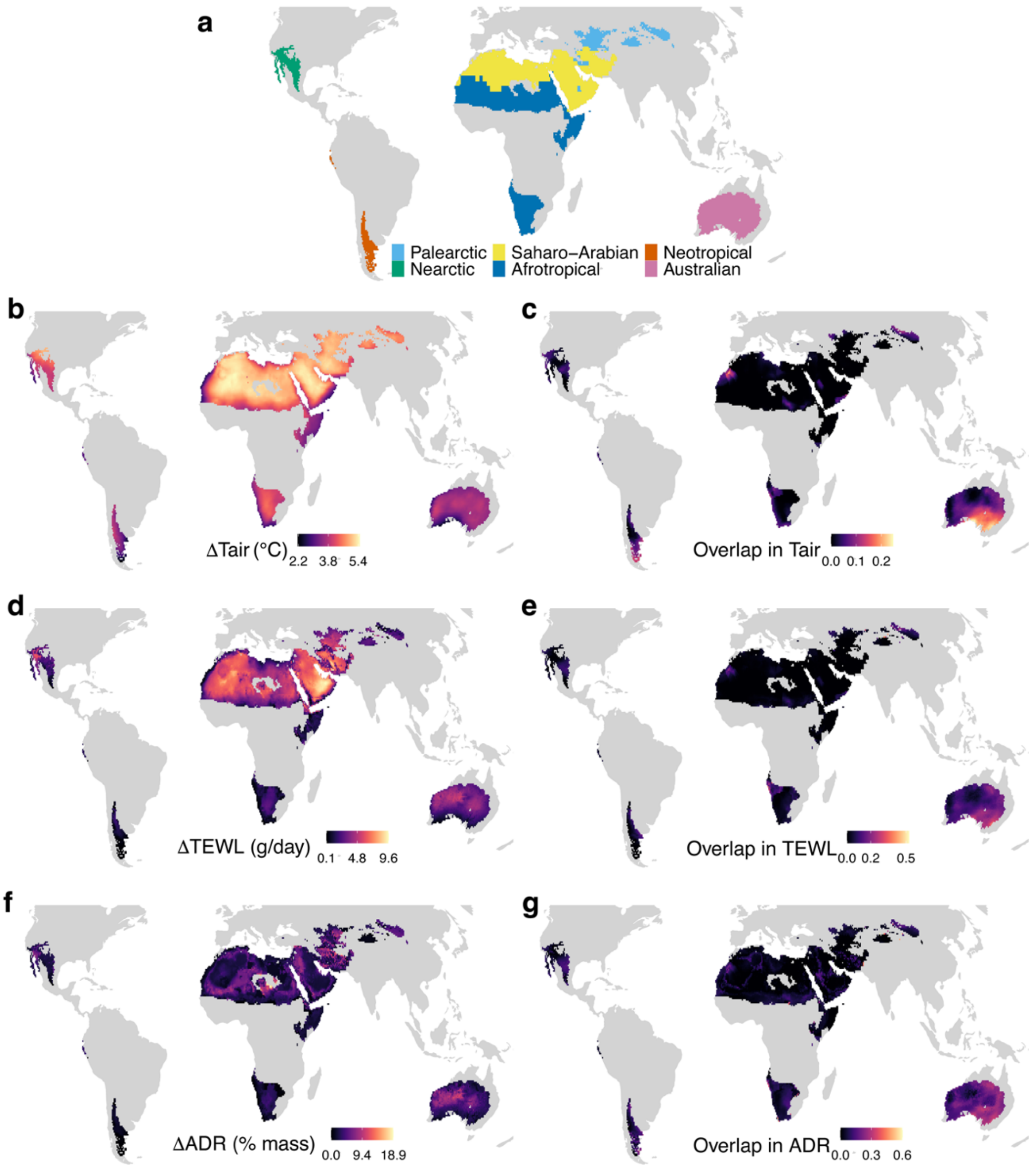

Supplementary Figure 7 Climate change impacts for desert birds when global mean temperatures are **4°C** warmer than pre-industrial values. The climate change impacts are shown as estimated changes in mean values (panels b, d, and f; "Δ" represent value changes; warmer colors indicate higher impact) and proportion of overlap between current and future values (panels c, e, and g; cooler colors indicate higher impact) of air temperature (Tair; °C), total evaporative water loss (TEWL; g/day) and acute dehydration risk (ADR; percent of body mass) during the hottest month (July for Northern Hemisphere, January for Southern Hemisphere). Panel a shows the locations of the six major realms containing warm deserts ("desert realms") and desert birds (bird species having ≥ 90% of their habitat within warm deserts). This figure assumed that the bird **actively shifts between open and shaded habitats** to minimize its water loss rate.

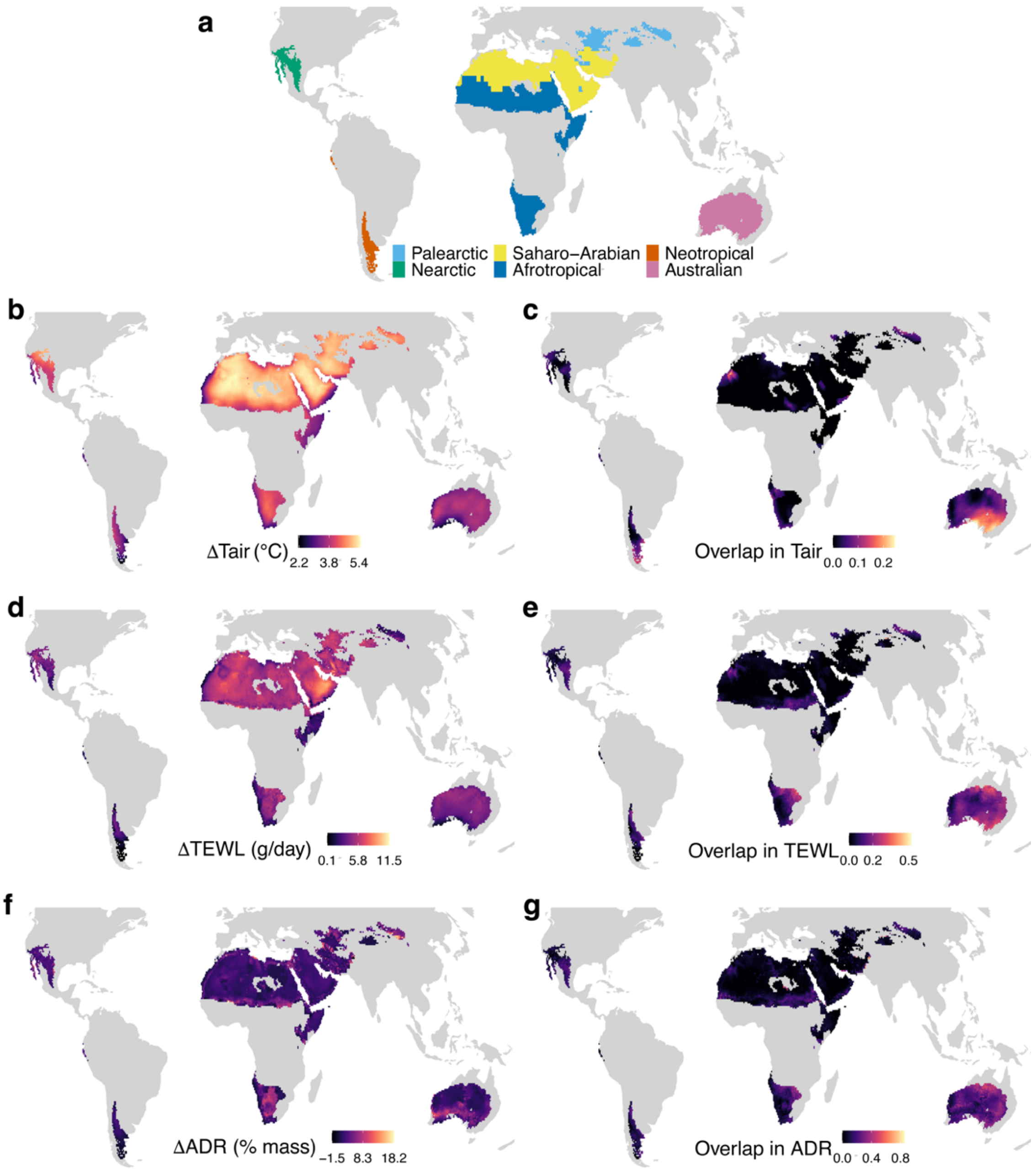

Supplementary Figure 8 Climate change impacts for desert birds when global mean temperatures are **4°C** warmer than pre-industrial values. The climate change impacts are shown as estimated changes in mean values (panels b, d, and f; "Δ" represent value changes; warmer colors indicate higher impact) and proportion of overlap between current and future values (panels c, e, and g; cooler colors indicate higher impact) of air temperature (Tair; °C), total evaporative water loss (TEWL; g/day) and acute dehydration risk (ADR; percent of body mass) during the hottest month (July for Northern Hemisphere, January for Southern Hemisphere). Panel a shows the locations of the six major realms containing warm deserts ("desert realms") and desert birds (bird species having ≥ 90% of their habitat within warm deserts). This figure assumed that the bird always stays **open habitat**.

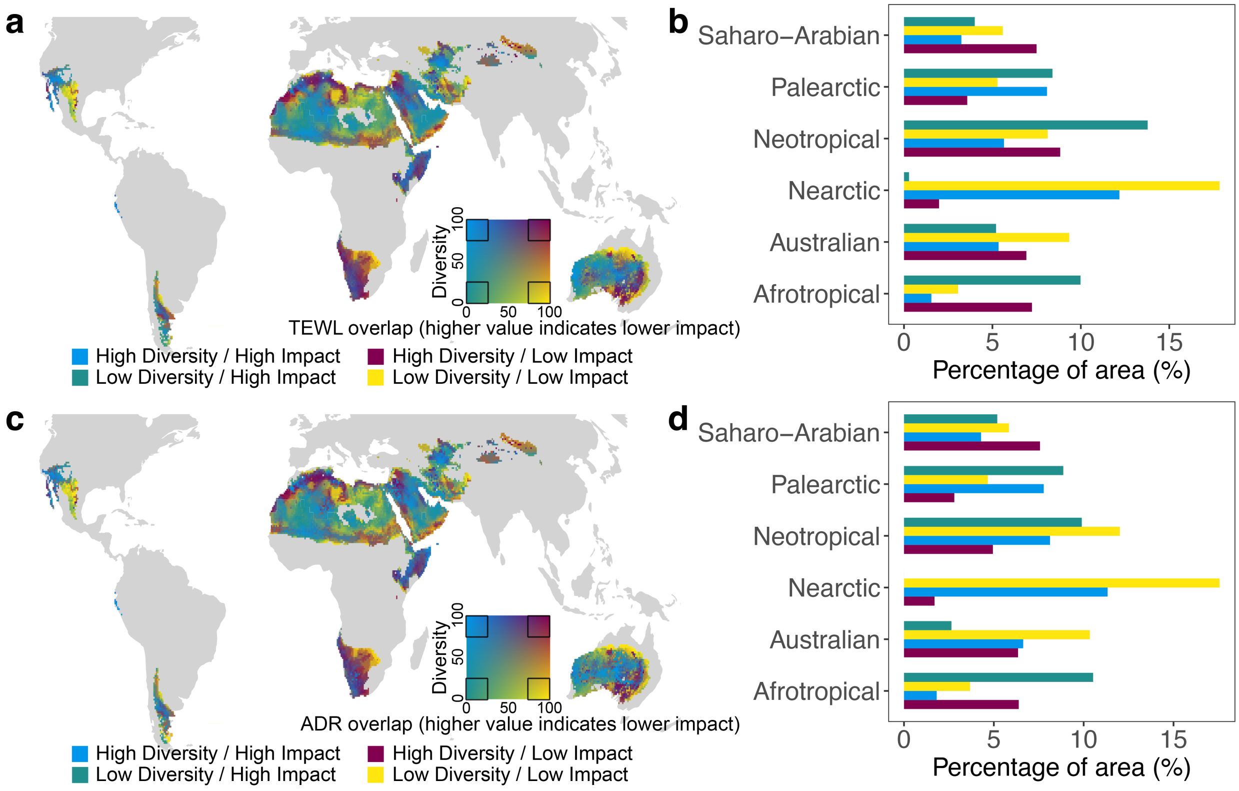

Supplementary Figure 9 Overlapping projections of climate change impacts and the distribution of desert bird diversity. Climate change impacts are measured as the proportion of overlap between current and future values of TEWL (panels a and b) or ADR (panels c and d) per pixel (higher overlap implies lower impact) when global mean temperatures are **2°C** warmer than pre-industrial values. We defined desert bird as bird species with ≥ 90% area of their global habitat area falling within warm deserts. Diversity is calculated as rarity-weighted species richness, where species are weighted by the size of their global Area of Habitat (AOH). Panels a and c are bivariate heatmaps that place each pixel along axes of TEWL/ADR overlap and diversity value (from 0 to 100 percentiles; mapping is done for each desert realm). Correspondingly, panels b and d show the percentages of area in each desert realm falling within the four categories defined by TEWL/ADR overlap and diversity value ("High" and "Low" are defined by whether the pixel value falls in the top or bottom 25% of all pixel values within that desert realm, respectively). For each pixel, we averaged the results for birds in three body mass categories (see Methods), weighted by the number of bird species in each category, to calculate TEWL and ADR values. This figure assumed that the bird always stays in **open habitat**.

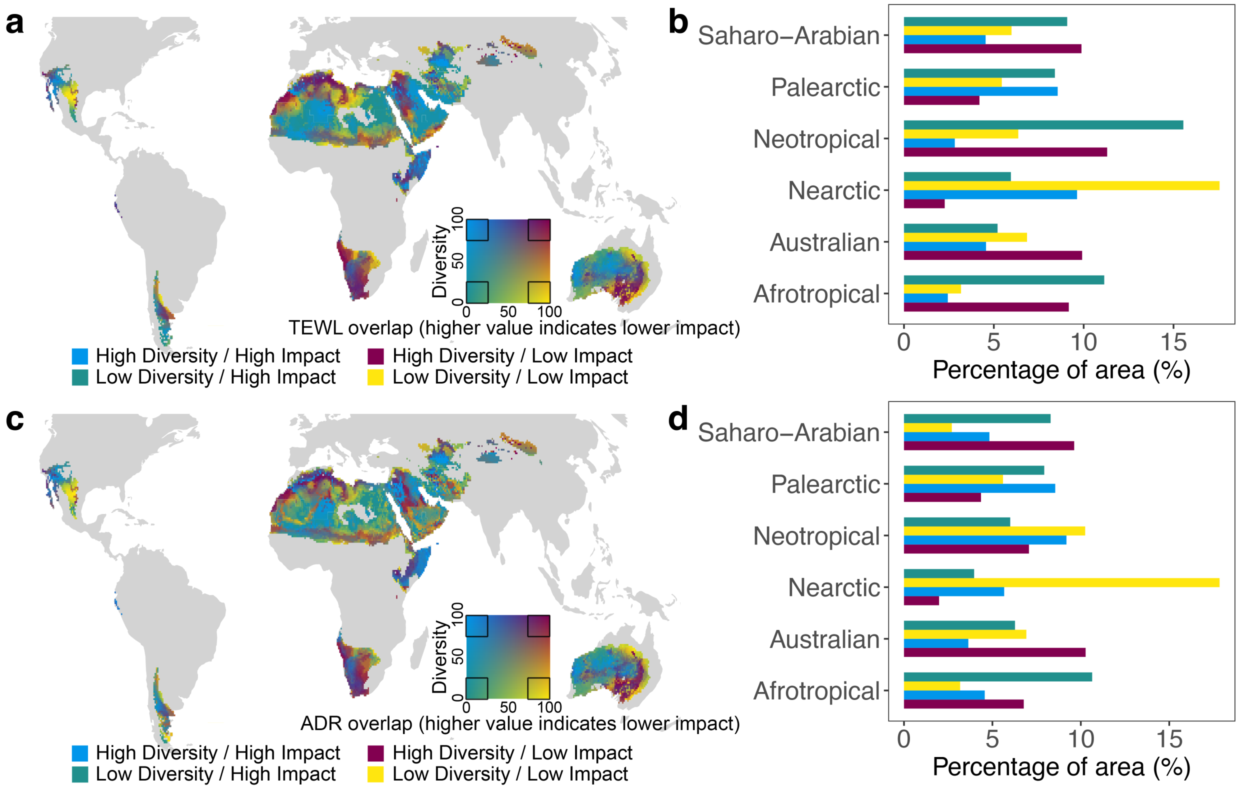

Supplementary Figure 10 Overlapping projections of climate change impacts and the distribution of desert bird diversity. Climate change impacts are measured as the proportion of overlap between current and future values of TEWL (panels a and b) or ADR (panels c and d) per pixel (higher overlap implies lower impact) when global mean temperatures are **4°C** warmer than pre-industrial values. We defined desert bird as bird species with ≥ 90% area of their global habitat area falling within warm deserts. Diversity is calculated as rarity-weighted species richness, where species are weighted by the size of their global Area of Habitat (AOH). Panels a and c are bivariate heatmaps that place each pixel along axes of TEWL/ADR overlap and diversity value (from 0 to 100 percentiles; mapping is done for each desert realm). Correspondingly, panels b and d show the percentages of area in each desert realm falling within the four categories defined by TEWL/ADR overlap and diversity value ("High" and "Low" are defined by whether the pixel value falls in the top or bottom 25% of all pixel values within that desert realm, respectively). For each pixel, we averaged the results for birds in three body mass categories (see Methods), weighted by the number of bird species in each category, to calculate TEWL and ADR values. This figure assumed that the bird actively **shifts between open and shaded habitats** to minimize its water loss rate.

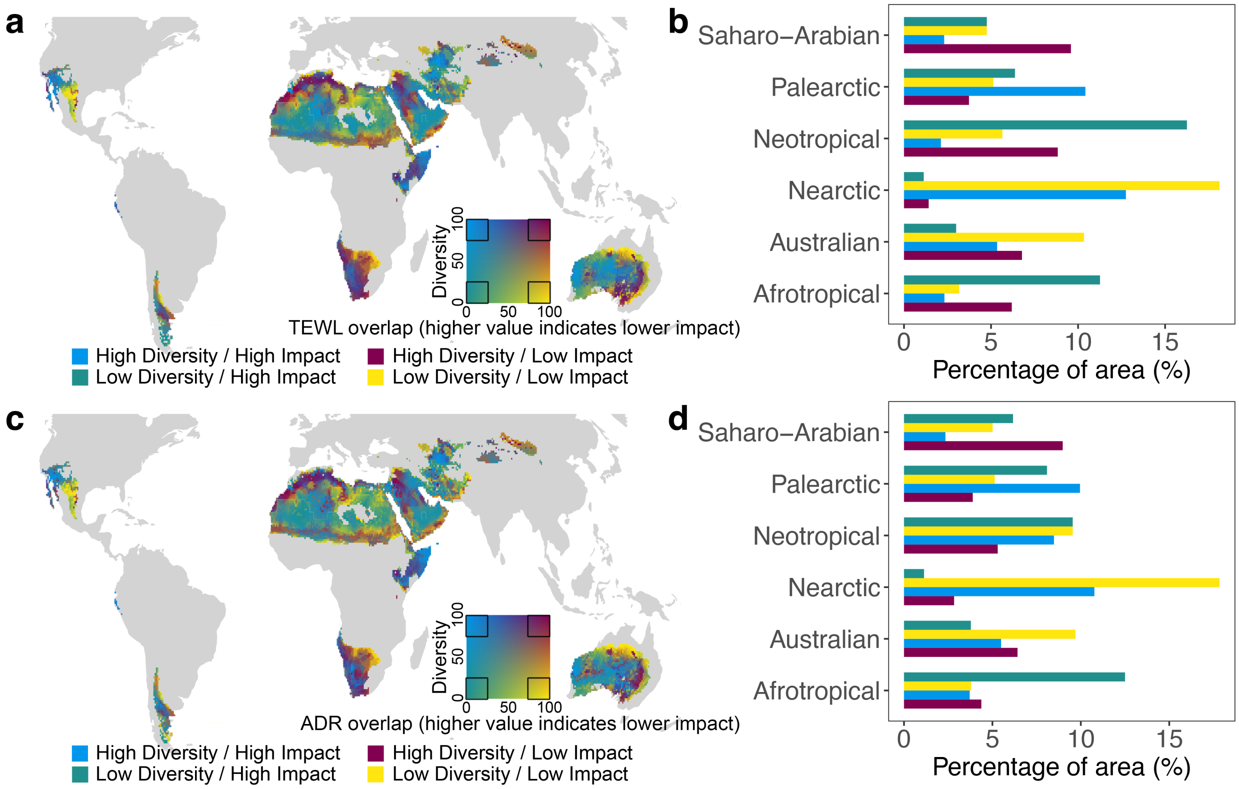

Supplementary Figure 11 Overlapping projections of climate change impacts and the distribution of desert bird diversity. Climate change impacts are measured as the proportion of overlap between current and future values of TEWL (panels a and b) or ADR (panels c and d) per pixel (higher overlap implies lower impact) when global mean temperatures are **4°C** warmer than pre-industrial values. We defined desert bird as bird species with ≥ 90% area of their global habitat area falling within warm deserts. Diversity is calculated as rarity-weighted species richness, where species are weighted by the size of their global Area of Habitat (AOH). Panels a and c are bivariate heatmaps that place each pixel along axes of TEWL/ADR overlap and diversity value (from 0 to 100 percentiles; mapping is done for each desert realm). Correspondingly, panels b and d show the percentages of area in each desert realm falling within the four categories defined by TEWL/ADR overlap and diversity value ("High" and "Low" are defined by whether the pixel value falls in the top or bottom 25% of all pixel values within that desert realm, respectively). For each pixel, we averaged the results for birds in three body mass categories (see Methods), weighted by the number of bird species in each category, to calculate TEWL and ADR values. This figure assumed that the bird always stays in **open habitat**.

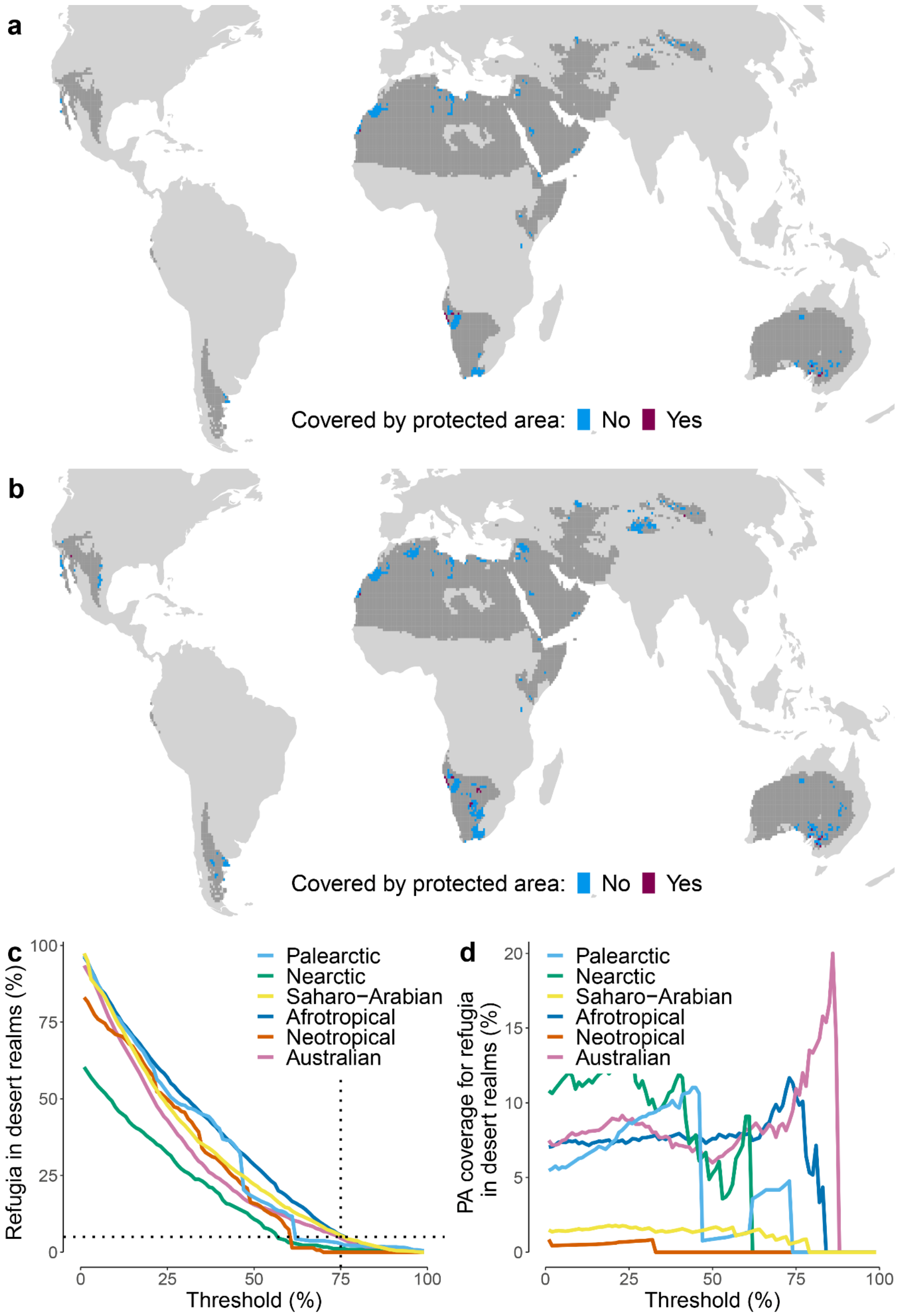

Supplementary Figure 12 Predicted locations of climate change refugia for desert birds in global warm deserts and their current protection status. The figure considers a climate change scenario that the global mean temperatures are **2°C** warmer than pre-industrial values. Panel a shows the refugia identified using a fixed threshold of 75th percentile (i.e. top 25%) for ADR overlap, TEWL overlap, and avian diversity, while panel b shows the refugia identified using a floating threshold such that at least 5% of desert area in each realm is identified as refugia (see text for details). Panel c shows the relationship between the threshold used and the percentage of desert area in each realm identified as refugia. Panel d shows the relationship between the threshold used and PA coverage for refugia identified in each realm. This figure assumed that the bird always stays in **open habitat**.

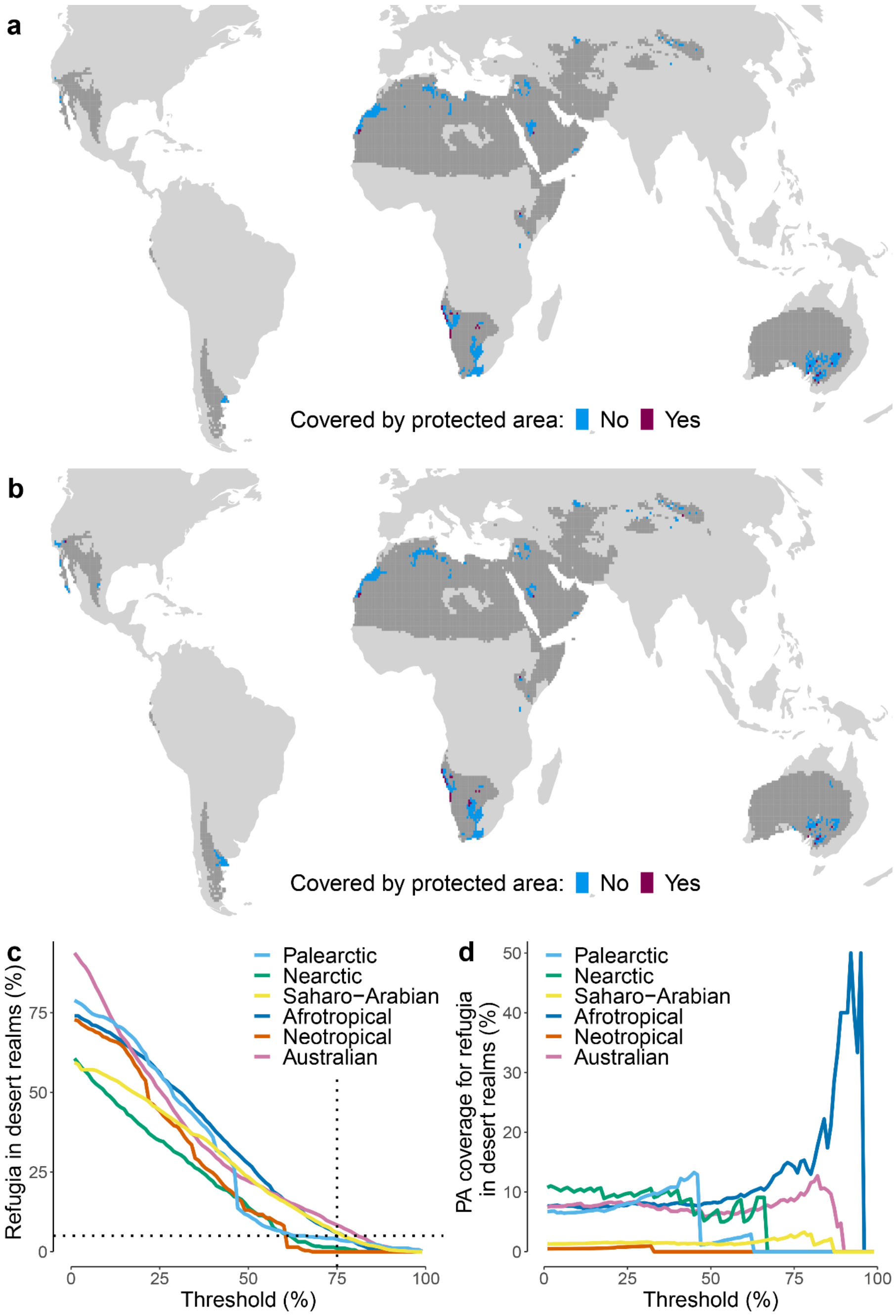

Supplementary Figure 13 Predicted locations of climate change refugia for desert birds in global warm deserts and their current protection status. The figure considers a climate change scenario that the global mean temperatures are **4°C** warmer than pre-industrial values. Panel a shows the refugia identified using a fixed threshold of 75th percentile (i.e. top 25%) for ADR overlap, TEWL overlap, and avian diversity, while panel b shows the refugia identified using a floating threshold such that at least 5% of desert area in each realm is identified as refugia (see text for details). Panel c shows the relationship between the threshold used and the percentage of desert area in each realm identified as refugia. Panel d shows the relationship between the threshold used and PA coverage for refugia identified in each realm. This figure assumed that the bird actively **shifts between open and shaded habitats** to minimize its water loss rate.

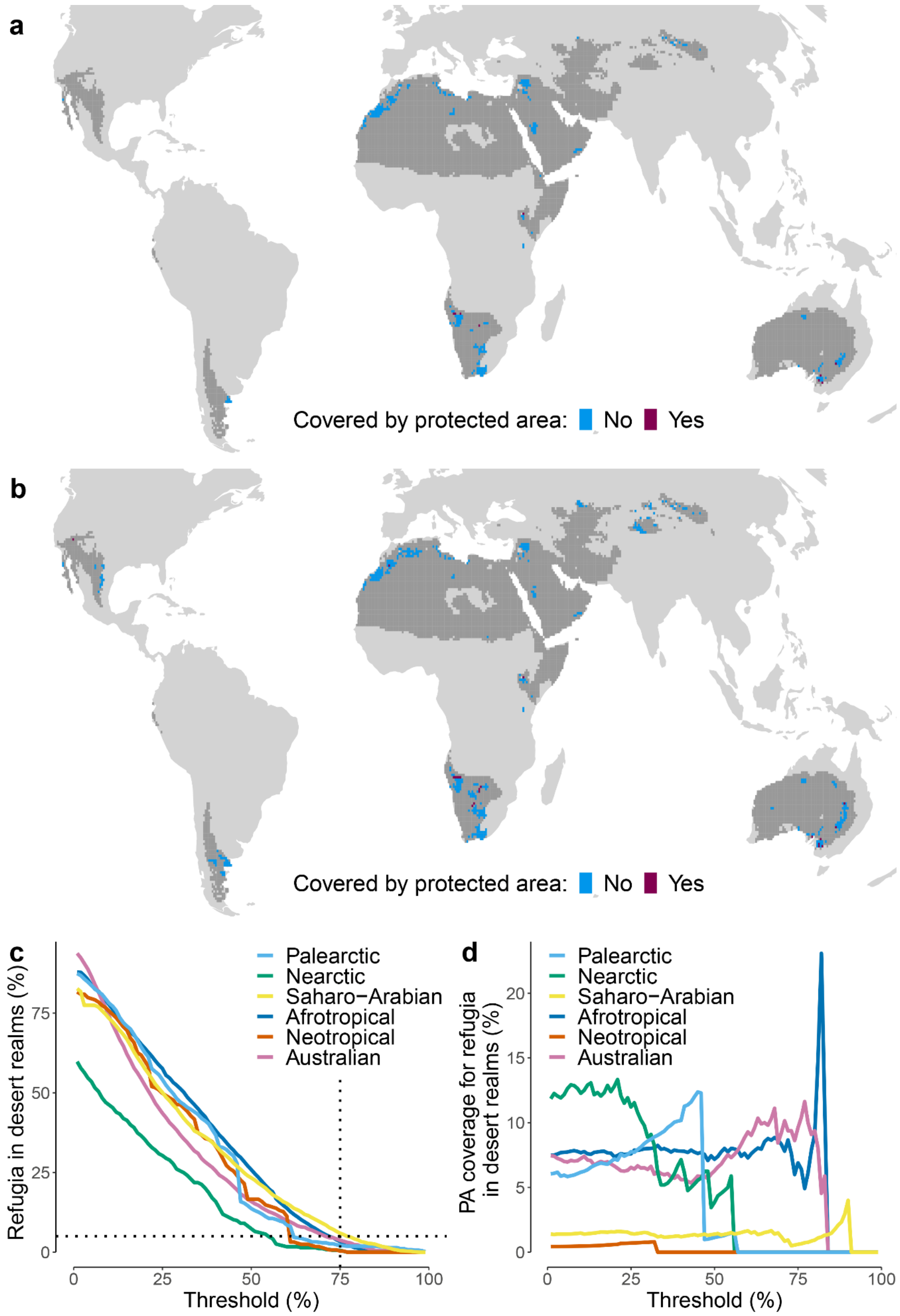

Supplementary Figure 14 Predicted locations of climate change refugia for desert birds in global warm deserts and their current protection status. The figure considers a climate change scenario that the global mean temperatures are **4°C** warmer than pre-industrial values. Panel a shows the refugia identified using a fixed threshold of 75th percentile (i.e. top 25%) for ADR overlap, TEWL overlap, and avian diversity, while panel b shows the refugia identified using a floating threshold such that at least 5% of desert area in each realm is identified as refugia (see text for details). Panel c shows the relationship between the threshold used and the percentage of desert area in each realm identified as refugia. Panel d shows the relationship between the threshold used and PA coverage for refugia identified in each realm. This figure assumed that the bird always stays in **open habitat**.

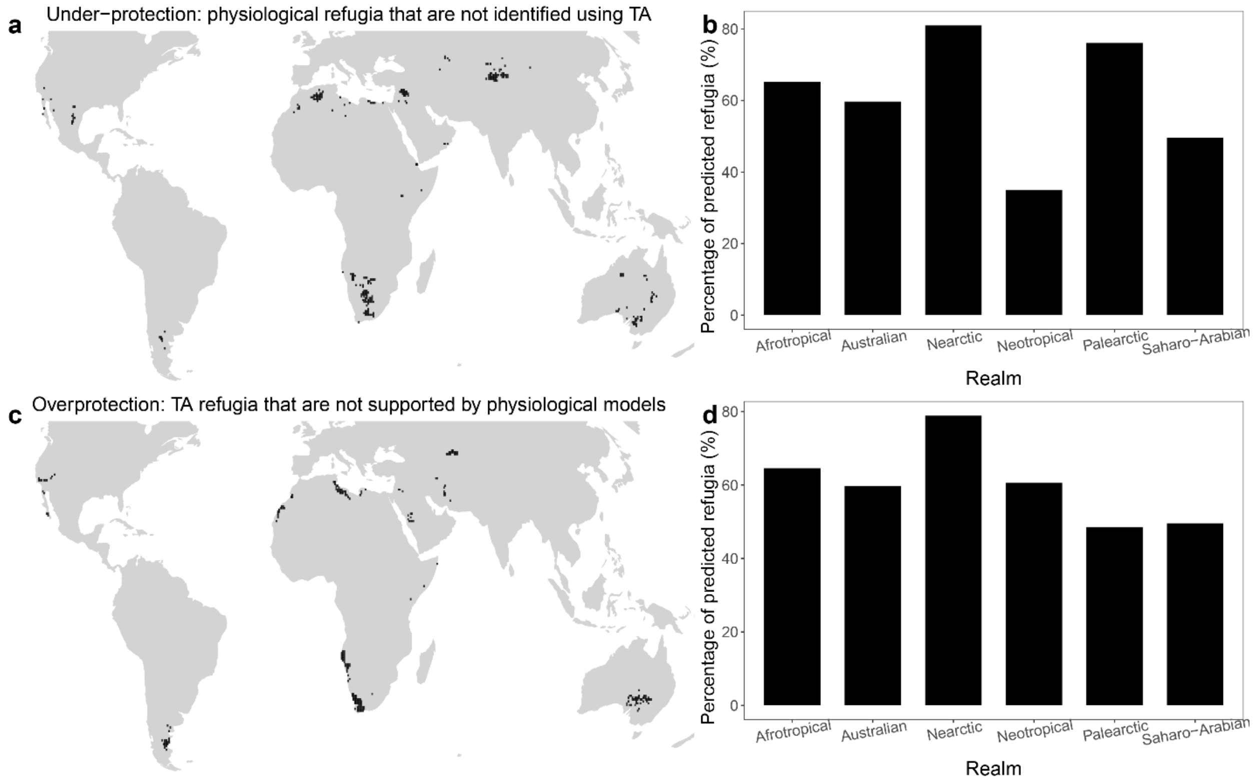

Supplementary Figure 15 Under-protection and overprotection when using air temperature as a proxy for physiological metric in identifying climate change refugia. The figure considers a climate change scenario that the global mean temperatures are **2°C** warmer than pre-industrial values. Panel a shows areas identified as refugia using physiological metrics that are not identified as refugia using Tair (i.e., "under-protection"); panel c shows areas identified as refugia using Tair that are not identified as refugia using physiological metrics (i.e., "overprotection"). Panel b and d show percentage of desert area in each desert realm where the "under-protection" and "overprotection" occurs, respectively. This figure assumed that the bird always stays in **open habitat**.

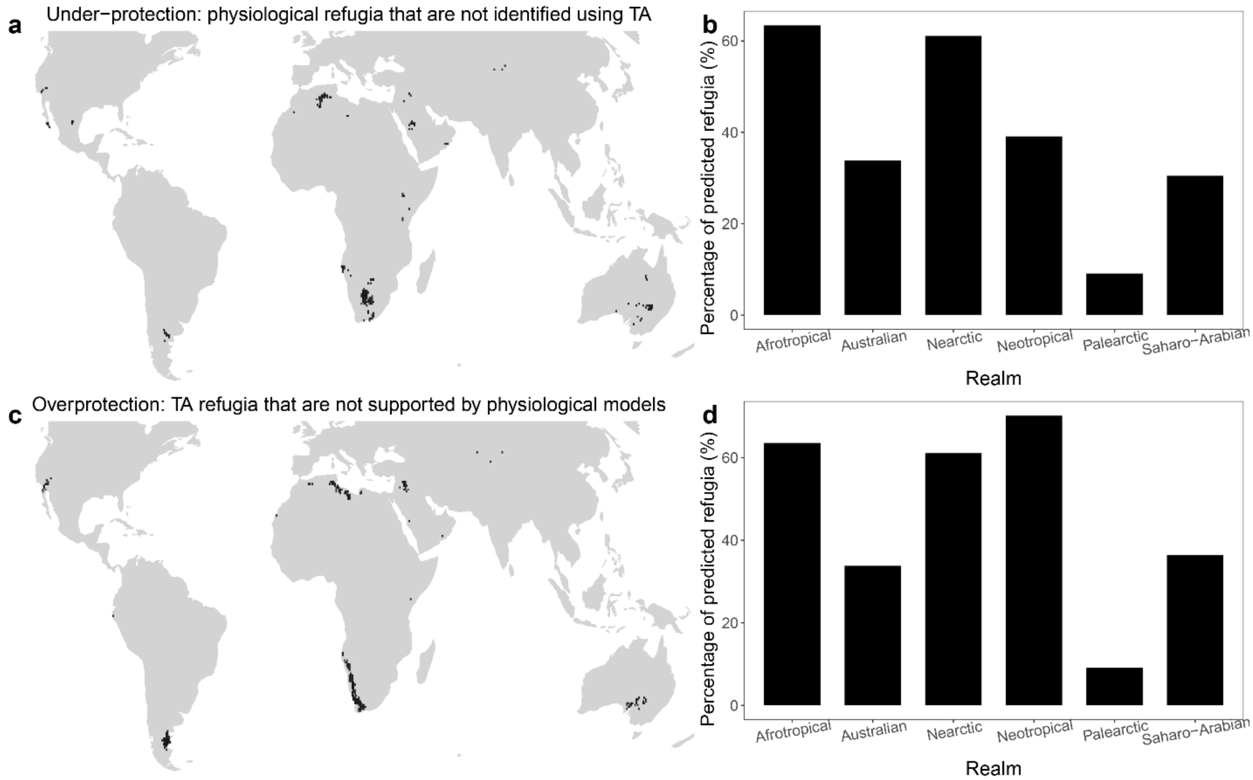

Supplementary Figure 16 Under-protection and overprotection when using air temperature as a proxy for physiological metric in identifying climate change refugia. The figure considers a climate change scenario that the global mean temperatures are **4°C** warmer than pre-industrial values. Panel a shows areas identified as refugia using physiological metrics that are not identified as refugia using Tair (i.e., "under-protection"); panel c shows areas identified as refugia using Tair that are not identified as refugia using physiological metrics (i.e., "overprotection"). Panel b and d show percentage of desert area in each desert realm where the "under-protection" and "overprotection" occurs, respectively. This figure assumed that the bird actively **shifts between open and shaded habitats** to minimize its water loss rate.

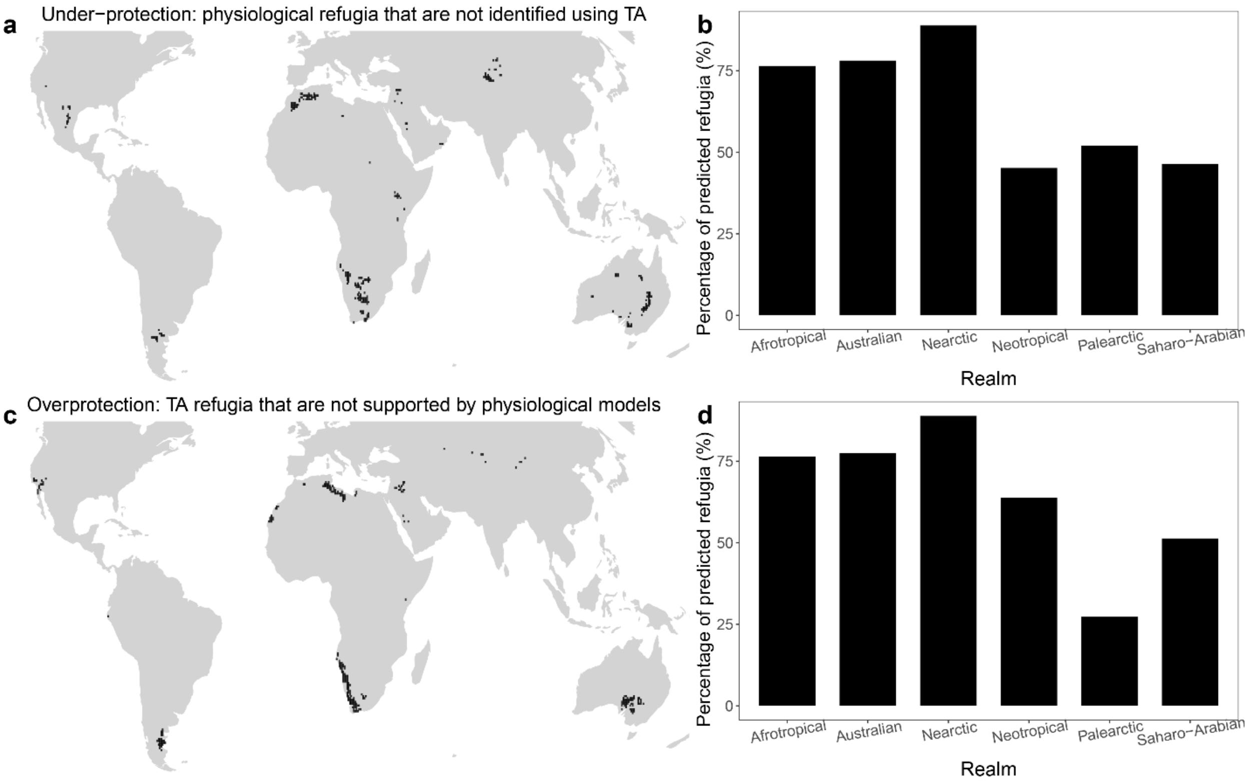

Supplementary Figure 17 Under-protection and overprotection when using air temperature as a proxy for physiological metric in identifying climate change refugia. The figure considers a climate change scenario that the global mean temperatures are **4°C** warmer than pre-industrial values. Panel a shows areas identified as refugia using physiological metrics that are not identified as refugia using Tair (i.e., "under-protection"); panel c shows areas identified as refugia using Tair that are not identified as refugia using physiological metrics (i.e., "overprotection"). Panel b and d show percentage of desert area in each desert realm where the "under-protection" and "overprotection" occurs, respectively. This figure assumed that the bird always stays in **open habitat**.

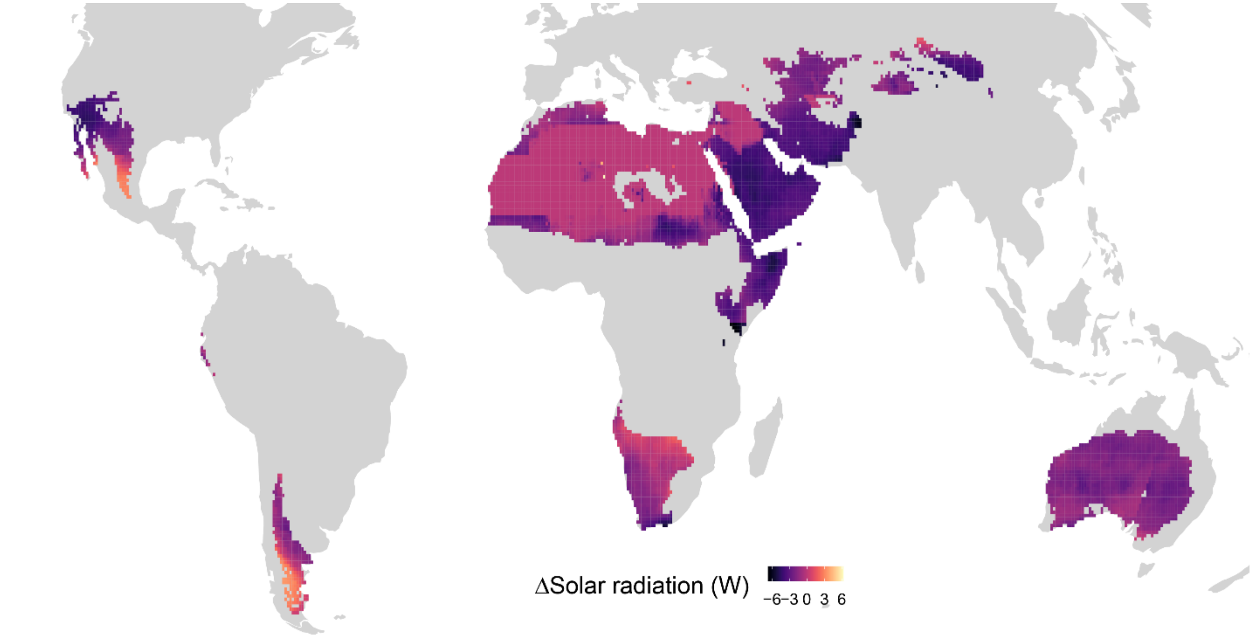

Supplementary Figure 18 Changes in mean solar radiation (W) during the hottest month (July for Northern Hemisphere, January for Southern Hemisphere) in global warm deserts. “Δ” represent value changes. The figure considers a climate change scenario that the global mean temperatures are 2°C warmer than pre-industrial values.

Supplementary References

1. Campbell, G. S. & Norman, J. *An introduction to environmental biophysics*. (Springer Science & Business Media, 2012).
