# Supplementary material for: Global patterns of climate change impacts on desert bird communities
Source: Nat Commun. 2023 Jan 13;14:211. doi: 10.1038/s41467-023-35814-8 (PMC9839677; doi:10.1038/s41467-023-35814-8)
Supplement: Supplementary file 2 — Description of Additional Supplementary Information [file 41467_2023_35814_MOESM2_ESM.docx]

File Name: Supplementary Data 1
Description: Desert bird species, body mass and size category. We defined desert bird species as bird species with more than 90% of their area of habitat falling within warm deserts (152 species). The body mass data is from AVONET^1^. We used the 33^th^ (19.6 g) and 66^th^ (48.6 g) percentiles of body masses of desert birds to divide them into three size categories: < 19.6 g; 19.6~48.6 g; > 48.6 g, then we modeled three birds weighed 13g, 39g and 185g to represent birds in these categories, respectively.

Reference: 1. Tobias, J. A. et al. AVONET: morphological, ecological and geographical data for all birds. Ecology Letters 25, 581–597 (2022).

File Name: Supplementary Data 2
Description: Parameter values for the customized endotherm model (“endoR_devel” in NicheMapR with modification). Note: we used size-related traits of Cactus Wren (*Campylorhynchus brunneicapillus*), which has a medium body mass (39 g) among desert birds. We also modeled smaller (13 g) and larger (185 g) birds representing small (0-33^th^ percentiles) and large (66-100^th^ percentiles) desert birds, and adjusting feather length and plumage depth in proportion to AMASS^1/3^ accordingly.

File Name: Supplementary Data 3
Description: Source data for Figure 3c.

File Name: Supplementary Data 4
Description: Source data for Figure 3d.

File Name: Supplementary Data 5

Description: Climate change impact data, bird diversity data, protected area coverage data generated in this study. Following are the descriptions for columns:

**Longitude, Latitude**: We used an equal-area projection: Eckert IV.

Realm: Name of desert realm

**dTA_2 (°C)**: Change in mean values of air temperature between current (1986-2015) and future values considering a climate change scenario that the global mean temperatures are 2°C warmer than pre-industrial values.

**dTA_4 (°C)**: Change in mean values of air temperature between current (1986-2015) and future values considering a climate change scenario that the global mean temperatures are 4°C warmer than pre-industrial values.

**dTEWL_open_2 (g/day)**: Change in mean values of total evaporative water loss between current (1986-2015) and future values considering a climate change scenario that the global mean temperatures are 2°C warmer than pre-industrial values. We assumed that a bird always stays in the open.

**dTEWL_open_4 (g/day)**: Change in mean values of total evaporative water loss between current (1986-2015) and future values considering a climate change scenario that the global mean temperatures are 4°C warmer than pre-industrial values. We assumed that a bird always stays in the open.

**dTEWL_shift_2 (g/day)**: Change in mean values of total evaporative water loss between current (1986-2015) and future values considering a climate change scenario that the global mean temperatures are 2°C warmer than pre-industrial values. We assumed that a bird actively shifts between open and shaded habitat to minimize its rate of water loss.

**dTEWL_shift_4 (g/day)**: Change in mean values of total evaporative water loss between current (1986-2015) and future values considering a climate change scenario that the global mean temperatures are 4°C warmer than pre-industrial values. We assumed that a bird actively shifts between open and shaded habitat to minimize its rate of water loss.

**dADR_open_2 (percent of body mass)**: Change in mean values of acute dehydration risk between current (1986-2015) and future values considering a climate change scenario that the global mean temperatures are 2°C warmer than pre-industrial values. We assumed that a bird always stays in the open.

**dADR_open_4 (percent of body mass)**: Change in mean values of acute dehydration risk between current (1986-2015) and future values considering a climate change scenario that the global mean temperatures are 4°C warmer than pre-industrial values. We assumed that a bird always stays in the open.

**dADR_shift_2 (percent of body mass)**: Change in mean values of acute dehydration risk between current (1986-2015) and future values considering a climate change scenario that the global mean temperatures are 2°C warmer than pre-industrial values. We assumed that a bird actively shifts between open and shaded habitat to minimize its rate of water loss.

**dADR_shift_4 (percent of body mass)**: Change in mean values of acute dehydration risk between current (1986-2015) and future values considering a climate change scenario that the global mean temperatures are 4°C warmer than pre-industrial values. We assumed that a bird actively shifts between open and shaded habitat to minimize its rate of water loss.

**oTA_2**: Proportion of overlap between current (1986-2015) and future values of air temperature considering a climate change scenario that the global mean temperatures are 2°C warmer than pre-industrial values.

**oTA_4**: Proportion of overlap between current (1986-2015) and future values of air temperature considering a climate change scenario that the global mean temperatures are 4°C warmer than pre-industrial values.

**oTEWL_open_2**: Proportion of overlap between current (1986-2015) and future values of total evaporative water loss considering a climate change scenario that the global mean temperatures are 2°C warmer than pre-industrial values. We assumed that a bird always stays in the open.

**oTEWL_open_4**: Proportion of overlap between current (1986-2015) and future values of total evaporative water loss considering a climate change scenario that the global mean temperatures are 4°C warmer than pre-industrial values. We assumed that a bird always stays in the open.

**oTEWL_shift_2**: Proportion of overlap between current (1986-2015) and future values of total evaporative water loss considering a climate change scenario that the global mean temperatures are 2°C warmer than pre-industrial values. We assumed that a bird actively shifts between open and shaded habitat to minimize its rate of water loss.

**oTEWL_shift_4**: Proportion of overlap between current (1986-2015) and future values of total evaporative water loss considering a climate change scenario that the global mean temperatures are 4°C warmer than pre-industrial values. We assumed that a bird actively shifts between open and shaded habitat to minimize its rate of water loss.

**oADR_open_2**: Proportion of overlap between current (1986-2015) and future values of acute dehydration risk considering a climate change scenario that the global mean temperatures are 2°C warmer than pre-industrial values. We assumed that a bird always stays in the open.

**oADR_open_4**: Proportion of overlap between current (1986-2015) and future values of acute dehydration risk considering a climate change scenario that the global mean temperatures are 4°C warmer than pre-industrial values. We assumed that a bird always stays in the open.

**oADR_shift_2**: Proportion of overlap between current (1986-2015) and future values of acute dehydration risk considering a climate change scenario that the global mean temperatures are 2°C warmer than pre-industrial values. We assumed that a bird actively shifts between open and shaded habitat to minimize its rate of water loss.

**oADR_shift_4**: Proportion of overlap between current (1986-2015) and future values of acute dehydration risk considering a climate change scenario that the global mean temperatures are 4°C warmer than pre-industrial values. We assumed that a bird actively shifts between open and shaded habitat to minimize its rate of water loss.

**RWR**: Rarity-weighted species richness.

**PA**: Whether the grid cell falls within existing protected areas.
