# Supplementary material for: Global patterns of climate change impacts on desert bird communities
Source: Nat Commun. 2023 Jan 13;14:211. doi: 10.1038/s41467-023-35814-8 (PMC9839677; doi:10.1038/s41467-023-35814-8)
Supplement: Supplementary file 8 — Reporting Summary [file 41467_2023_35814_MOESM8_ESM.pdf]

nature portfolio

Corresponding author(s): Liang Ma

Last updated by author(s): Dec 29, 2022

Reporting Summary

Nature Portfolio wishes to improve the reproducibility of the work that we publish. This form provides structure for consistency and transparency in reporting. For further information on Nature Portfolio policies, see our Editorial Policies and the Editorial Policy Checklist.

Statistics

For all statistical analyses, confirm that the following items are present in the figure legend, table legend, main text, or Methods section.

n/a	Confirmed	
x		The exact sample size (*n*) for each experimental group/condition, given as a discrete number and unit of measurement
x		A statement on whether measurements were taken from distinct samples or whether the same sample was measured repeatedly
	x	The statistical test(s) used AND whether they are one- or two-sided<br>*Only common tests should be described solely by name; describe more complex techniques in the Methods section.*
x		A description of all covariates tested
	x	A description of any assumptions or corrections, such as tests of normality and adjustment for multiple comparisons
x		A full description of the statistical parameters including central tendency (e.g. means) or other basic estimates (e.g. regression coefficient) AND variation (e.g. standard deviation) or associated estimates of uncertainty (e.g. confidence intervals)
	x	For null hypothesis testing, the test statistic (e.g. *F*, *t*, *r*) with confidence intervals, effect sizes, degrees of freedom and *P* value noted<br>*Give P values as exact values whenever suitable.*
x		For Bayesian analysis, information on the choice of priors and Markov chain Monte Carlo settings
x		For hierarchical and complex designs, identification of the appropriate level for tests and full reporting of outcomes
	x	Estimates of effect sizes (e.g. Cohen's *d*, Pearson's *r*), indicating how they were calculated

Our web collection on statistics for biologists contains articles on many of the points above.

Software and code

Policy information about availability of computer code

Data collection	Maps of area of habitat, warm deserts and rarity-weighted richness were created using Google Earth Engine (2021). The microclimate data was created using a microclimate model in an R (version 4.0.3) package -- NicheMapR (version 3.1.0). We calculated the overlapped estimated area of kernel density estimations for current and future values of TEWL and ADR using the “overlap” function in R package “overlapping” (version 1.8). We used the R package “rcompanion” (version 2.4.16) to calculate the Epsilon-Squared as the effect size statistic. Custom codes developed in this study have been deposited in Zenodo: https://doi.org/10.5281/zenodo.7088572
Data analysis	All statistical analyses were performed in R (version 4.0.3).

For manuscripts utilizing custom algorithms or software that are central to the research but not yet described in published literature, software must be made available to editors and reviewers. We strongly encourage code deposition in a community repository (e.g. GitHub). See the Nature Portfolio guidelines for submitting code & software for further information.

Data

Policy information about availability of data

All manuscripts must include a data availability statement. This statement should provide the following information, where applicable:

- Accession codes, unique identifiers, or web links for publicly available datasets
- A description of any restrictions on data availability
- For clinical datasets or third party data, please ensure that the statement adheres to our policy

Habitat classification scheme of IUCN (version 3.1): https://github.com/Martin-Jung/Habitatmapping; WorldClim 2: http://www.worldclim.com/version2; An updated map of Wallace's zoogeographic regions of the world: https://macroecology.ku.dk/resources/wallace; TerraClimate: https://www.climatologylab.org/terraclimate.html; Maps of the spatial distribution of bird species from BirdLife: http://datazone.birdlife.org/species/requestdis; World Database on Protected Areas (WDPA): www.protectedplanet.net; California Basin Characterization Model dataset: http://climate.calcommons.org/bcm.
Source data are provided for Figure 3c (Supplementary Data 3) and Figure 3d (Supplementary Data 4). Climate change impact data, bird diversity data, protected area coverage data generated in this study are provided as Supplementary Data 5.

Human research participants

Policy information about studies involving human research participants and Sex and Gender in Research.

Reporting on sex and gender	No sex- or gender-based analyses have been performed in our study as sex- or gender-based traits required for parameterizing the biophysical models are largely unavailable for desert birds.
Population characteristics	See above.
Recruitment	See above.
Ethics oversight	See above.

Note that full information on the approval of the study protocol must also be provided in the manuscript.

Field-specific reporting

Please select the one below that is the best fit for your research. If you are not sure, read the appropriate sections before making your selection.

☐ Life sciences ☐ Behavioural & social sciences ☒ Ecological, evolutionary & environmental sciences

For a reference copy of the document with all sections, see nature.com/documents/nr-reporting-summary-flat.pdf

Ecological, evolutionary & environmental sciences study design

All studies must disclose on these points even when the disclosure is negative.

Study description	We combined climate change projections with physiological models and species distributions to predict physiological impacts of climate change on desert birds globally.
Research sample	We modeled desert bird species, which were defined as bird species with more than 90% of their area of habitat falling within warm deserts (152 species). Model projections were performed at all sites in the area of habitat for each species.
Sampling strategy	This is not applicable because we did not actually sample any species in this modeling study.
Data collection	The authors downloaded the data used in this study from online source (see Data Availability and Author Contributions Statement).
Timing and spatial scale	We used historical monthly climate data from the TerraClimate dataset (50 km spatial resolution) for our simulations, which provides maximum temperature, minimum temperature, precipitation, soil moisture, vapor pressure, downward surface shortwave radiation and wind-speed. The 'micro_terra' function of NicheMapR disaggregates the air temperature data to hourly following methods described in Kearney and Porter (2017), and then computes hourly relative humidity from the vapor pressure and air temperature. It also computes cloud clover from the ratio between observed shortwave radiation and predicted clear sky radiation. We extracted monthly climate data for global warm deserts for what is typically the hottest month (July and January for Northern and Southern Hemispheres, respectively) for 1986-2015 and for pseudo years 1986-2015 commensurate with two different climate futures: (1) when global mean temperatures are 2°C warmer than pre-industrial values, and (2) when global mean temperatures are 4°C above pre-industrial values. The climate change scenarios were derived from 23 CMIP5 global climate models as described in Qin et al.

	2020.
Data exclusions	No data were excluded from the analyses.
Reproducibility	No experiments were conducted in this study.
Randomization	This is not applicable because we did not actually sample any species in this modeling study.
Blinding	Blinding is not relevant to our study because no controlled experiments were conducted.

Did the study involve field work? ☐ Yes ☒ No

Reporting for specific materials, systems and methods

We require information from authors about some types of materials, experimental systems and methods used in many studies. Here, indicate whether each material, system or method listed is relevant to your study. If you are not sure if a list item applies to your research, read the appropriate section before selecting a response.

Materials & experimental systems

n/a	Involved in the study
☒	☐ Antibodies
☒	☐ Eukaryotic cell lines
☒	☐ Palaeontology and archaeology
☒	☐ Animals and other organisms
☒	☐ Clinical data
☒	☐ Dual use research of concern

Methods

n/a	Involved in the study
☒	☐ ChIP-seq
☒	☐ Flow cytometry
☒	☐ MRI-based neuroimaging
